# Latest Trends in Electrochemical Sensors for Neurotransmitters: A Review

**DOI:** 10.3390/s19092037

**Published:** 2019-04-30

**Authors:** Zahra Tavakolian-Ardakani, Oana Hosu, Cecilia Cristea, Mohammad Mazloum-Ardakani, Giovanna Marrazza

**Affiliations:** 1 Department of Chemistry “Ugo Schiff”, University of Florence, Via della Lastruccia 3, 50019 Sesto Fiorentino (Fi), Italy; tavakolianz@yahoo.com (Z.T.-A); hosuoanaalexandra@gmail.com (O.H.); 2 Department of Chemistry, Faculty of Science, Yazd University, Yazd 89195-741, Iran; mazloum@yazd.ac.ir; 3 Department of Analytical Chemistry, Faculty of Pharmacy, “Iuliu Haţieganu” University of Medicine and Pharmacy, 400349 Pasteur 4 Cluj-Napoca, Romania; ccristea@umfcluj.ro; 4 Instituto Nazionale Biostrutture e Biosistemi (INBB), Unit of Florence, Viale delle Medaglie d’Oro 305, 00136 Roma, Italy

**Keywords:** neurotransmitter, electrochemical, sensor, enzyme

## Abstract

Neurotransmitters are endogenous chemical messengers which play an important role in many of the brain functions, abnormal levels being correlated with physical, psychotic and neurodegenerative diseases such as Alzheimer’s, Parkinson’s, and Huntington’s disease. Therefore, their sensitive and robust detection is of great clinical significance. Electrochemical methods have been intensively used in the last decades for neurotransmitter detection, outclassing more complicated analytical techniques such as conventional spectrophotometry, chromatography, fluorescence, flow injection, and capillary electrophoresis. In this manuscript, the most successful and promising electrochemical enzyme-free and enzymatic sensors for neurotransmitter detection are reviewed. Focusing on the activity of worldwide researchers mainly during the last ten years (2010–2019), without pretending to be exhaustive, we present an overview of the progress made in sensing strategies during this time. Particular emphasis is placed on nanostructured-based sensors, which show a substantial improvement of the analytical performances. This review also examines the progress made in biosensors for neurotransmitter measurements in vitro, in vivo and ex vivo.

## 1. Introduction

Neurotransmitters (NTs) are endogenous chemical messengers which neurons use to communicate with each other, to act on muscle cells or to stimulate a response by glandular cells. In the 1921s, the Nobel Prize winner, Otto Loewi discovered the first known neurotransmitter—acetylcholine—through his experiments on the nervous regulation of cardiac activity [1]. Since the discovery of the first neurotransmitter, more than one hundred chemical messengers involved in neuronal transmissions have been revealed [2]. The large number of recognized neurotransmitters has made it essential to classify these chemical molecules, so as to simplify their study. There are several classification criteria based on physiological function, either excitatory or inhibitory, molecular structure and mode of action, either direct or as a neuromodulator. The most common is the one that distinguishes neurotransmitters according to the class of molecules they belong to. The main classes of molecules to which the human neurotransmitters belong are: amino acids (i.e., glutamic acid and tyrosine), biogenic amines (i.e., epinephrine, nor-epinephrine, dopamine and serotonin) and soluble gases (i.e., nitric oxide and hydrogen sulfide) [3]. All those neurotransmitters that cannot be grouped in any of the previous classes, such as acetylcholine and choline, fall under the heading “other”. NTs play an important role in many of the brain functions, such as behavior and cognition, cardiovascular, renal, and hormonal functions systems along with establishing human brain-body integration. They affect and control heart rate and muscle tone, as well as adjustment of learning, sleeping, memory, consciousness, mood and appetite. Changes in the concentration of NTs in the central nervous system have been correlated with numerous psychotic (schizophrenia, depression, dementia, etc.), neurodegenerative diseases (Alzheimer’s, Parkinson’s, Huntington’s disease, autism, epilepsy, etc.), and physical illnesses (glaucoma, arrhythmias, thyroid hormone shortage, congestive heart damage, sudden infant death syndrome, dejection and anguish, etc.) [4]. The most important neurotransmitters and their characteristics are reported in Table 1.

Therefore, the quantitative detection of the neurotransmitter in different human fluids is of great importance for diagnosis, monitoring disease state and therapeutic interventions. Various analytical methods to analyze neurotransmitters are reported, such as mass spectroscopy, fluorimetry, chemiluminescence, chromatography and capillary electrophoresis. However, these techniques are expensive, performed by highly trained personnel with long waiting times before the analysis response is obtained and delivered to the patient. As such, these techniques are not suitable for on-site monitoring.

In order to improve patient care, clinical laboratories have been challenged to develop new tests that are reliable, cost-effective and accurate, and to optimize existing protocols by making them faster and more efficient. A rapid and accurate screening of health conditions represents a key step in order to identify the sign of symptoms of a disease or of an altered physiological process although their effects are yet to come forward.

The use of electrochemical sensors for neurotransmitter determination represents a perfect analytical approach considering their low cost and the little time required for the analysis, as well as the possibility to detect two or more analytes simultaneously [4,5,6,7,8,9]. Moreover, the sensors are suitable for on-site detection or even imagined as a routine chair-side test represented by a point-of-care testing (POCT) device used by untrained personal. Due to the similarities of biogenic amines, it is mandatory to use selective methods especially in biological fluids in which all those neurotransmitters are present. Electrochemical biosensors, which associate a bioelement such as an enzyme [5], increase the selectivity issues and allow non-electroactive NT analysis.

The aim of the present review is to emphasize recent advances in electrochemical sensors for NT detection using direct measurements of electroactive neurotransmitters and enzyme-mediated reactions, without pretending of being exhaustive; therefore, we mainly selected recent research articles (from 2010 to 2019) having two commonalities: the use of nanomaterials in the sensors’ development and a clear focus on NT analysis. In addition, this review examines the progress made in biosensors for NTs in vitro, in vivo and ex vivo measurements. 

## 2. Electrochemical Sensors

With respect to other transduction systems (optical, piezoelectric, acoustic, gravimetric, magnetic, calorimetric), electrochemical sensors are highly sensitive, inexpensive, easy-to-use, portable and compatible with microfabrication technologies. Therefore, they have found application in a large number of clinical analyses. 

Neurological biomarkers are often present in biological fluids at ultra-low levels and require ultrasensitive detection methods. Different strategies have been employed to realize the modification of electrode surfaces for improving the selectivity, sensitivity, and accuracy of the (bio)sensors. 

Conductive polymers (CPs) and their composites have been extensively used to modify the electrode surfaces and several excellent reviews have been published [6,7,8]. The polymer is usually prepared in situ for electrodeposition offering the possibility to control the polymer thickness and to use non-conventional electrode geometries. However, the integration between the electron transfer mechanism at the electrode surface and the subsequent charge transport through the polymer backbone has to be considered for efficiency of the sensors. Polymeric films are useful for the NTs determination in the presence of interferents such as ascorbic acid, uric acid as they are eliminated by electrostatic repulsion.

Several examples are reported in the literature, for instance, the use of monomers as aniline [9,10,11], hydroxybenzoic acid [12], 3-hydroxyphenylacetic acid [13], xanthurenic acid [14], gallic acid [15], pyrrole [16], pyrene [17], methylene blue [18,19,20], among others [21]. Some CPs (i.e., polypyrrole, polyaniline, polymethylene blue) combined with biomolecules having cell adhesion functionality were electrodeposited with great precision onto microelectrode sites [22]. A recent review exhaustively discussing CPs-based sensors applied to neurotransmitter detection is available [3].

An alternative approach has been used by coating of electrode surface with a Nafion^®^ polymeric membrane. Nafion^®^ consists of a tetrafluoroethylene main chain with perfluoroether side chains terminated with a sulfonic acid group. Several studies in the literature [23,24,25] demonstrate the increase the selectivity of Nafion^®^-coated sensors in the determination of catecholamines in biological fluids minimizing the effect of some endogenous interferences (ascorbic acid and uric acid).

An attractive alternative procedure to improve the sensitivity stability, selectivity of electrochemical (bio)sensors is fabricating the modified electrodes based on the unique catalytic property of nanomaterials. 

They are materials are characterized by nanometric size (1–100 nm) in one or more dimensions. The resulting physical, chemical, mechanical, magnetic, and optical properties are very different from those of bulk materials. Due to their excellent physical and chemical properties, including high conductivity, relative inertness, wide voltage range, and fast heterogeneous electron transfer, carbon- based nanomaterias electrodes, including graphene, carbon nanotubes (CNTs), and carbon black, have been widely used.

Graphene is defined by the International Union for Pure and Applied Chemistry (IUPAC) as “a single carbon layer of graphite structure, describing its nature by analogy to a polycyclic aromatic hydrocarbon of quasi-infinite size”. It presents interesting electronic and structural characteristics such as high surface area, high thermal and electrical conductivity. This nanomaterial has recently attracted enormous attention and has been extensively applied in the last years in several sectors including electronics, nanomedicine, and electroanalysis as well. However, pristine graphene is not very useful for biological applications because it has a tendency to aggregate. An approach to solve this problem is to functionalize the graphene nanosurface. The most used functionalized structure derived from graphene is graphene oxide (GO). Several graphene-based sensors have been reported for various neurotransmitters measurements in biological samples [26,27,28,29,30,31,32,33,34,35].

CNTs represent another smart nanomaterial group with geometrical, electronic and chemical properties. CNTs are fullerene-like structures that can be single-walled (SWNTs) or multi-walled (MWNTs) shaped. SWNTs are cylindrical graphite sheets of 0.5–1 nm diameter capped by hemispherical ends, while MWNTs comprise several concentric cylinders of these graphitic shells with a layer spacing of 0.3–0.4 nm. CNTs are well assembled and have a greater area and strength, and overall enhanced chemical and thermal stability. Their outstanding properties are exploited for numerous applications in both therapeutic and diagnostic applications [36].

They show a large surface area and an electrocatalytic effect that have been used in developing electrochemical sensors. The majority of them have been obtained by modifying carbon electrode surfaces with a dispersion of CNTs in polymers or solvents, thus, increasing the sensitivity of the analysis by orders of magnitude with respect to the bare electrode surface. Numerous CNT-based sensors are reported in literature for neurotransmitter detection [37,38,39,40,41,42,43,44,45].

Carbon black (CB) is a form of amorphous carbon that has an extremely high surface area to volume ratio, and it has been one of the first nanomaterials for sensing applications for its electrochemical properties. The modification of electrode surface has been performed by drop casting as well as including CB in the ink of the screen-printed electrodes. CB-graphene composites and combined with chitosan have also been used for electrochemical determination of dopamine and epinephrine [46]. Yang et al. have reviewed in depth carbon nanomaterial sensors for biogenic amines detection [47].

Among various nanomaterials, nanoparticles, obtained from metals, semiconductor, carbon and polymeric materials, play a special role; they have been extensively applied in sensor development as quantification tags, immobilization substrates, for signal amplification, and as carriers. 

Metallic NPs are the most widely used as nanomaterial-modified electrochemical transducers, due to their valuable electrochemical properties (oxidation or reduction current). The fabrication of a metallic nanoparticle-based sensor is usually performed by drop casting and the electrochemical reduction of metal salts. The NPs present different dimensions and consequently different sensitivity depending on how they have been produced. 

The versatile applications of gold NPs (AuNPs) are strongly related to their easy chemical and biological modification. Particularly, the high affinity of thiols towards the surfaces of noble metals also facilitate the biofunctionalization of these metallic nanostructure by utilizing the broadly developed and well-defined organic surface chemistry for biological modifications [48,49]. A variety of other metals such as silver, copper, platinum, cobalt, nickel, or iron have been used in neurotransmitter sensing [50,51]. 

Magnetic particles (MNPs) with high magnetic susceptibilities are currently being used for sensor and biosensor development because they offer benefits such as a large surface area and the easy immobilization of enzymes. MNPs have been also used as peroxidase mimics for a sensitive choline biosensor [52].

In recent years, scientists have demonstrated that combining electronic properties of nanostructured conducting polymers with organic and inorganic units at the molecular scale leads to the development of advanced materials with well-controlled composition and properties. Hybrid materials provide rapid and accurate sensing due to their selectivity, high sensitivity, more active sites, homogeneity, and strong adherence to the electrode surface [6,53,54,55,56].

## 3. Non-Enzymatic Electrochemical Sensors

Non-enzymatic electrochemical sensors (also called enzyme-free or direct electrochemical sensors) have been widely used for the determination of NTs as some of them are electroactive and measuring their concentration in biological samples is of high importance for clinical diagnosis. Among these can be mentioned monoamines like dopamine, norepinephrine, serotonin [57,58,59].

The following section describes in detail several different types of neurotransmitter sensors for direct detection of neurotransmitters, which are presented considering the classification according to the target neurotransmitter. Direct sensing of NTs based on nanocomposite-modified electrodes is a good strategy for highly sensitive detection, but the presence of interferent molecules poses difficulties so only a few approaches are efficient for analysis in biological fluids.

Considering the abundant and highly scattered information in the literature, this review summarizes the recent research works about electroactive neurotransmitter detection as can be seen in Table 2. Approaches are compared with respect to electrode modifiers, linear range, LODs, sensitivity, electrochemical technique, electrolyte, real samples, storage stability, interferences and important observations relating to the sensors.

### 3.1. Amino Acid Neurotransmitters

The major group of neurotransmitters present in the central nervous system are amino acids. They provide the majority of inhibitory and excitatory neurotransmission in the body. 

#### Glutamate

Glutamate is one of the prominent neurotransmitters in the mammalian central nervous system [60,61,62] where nearly 90% of all neurons use this amino acid as a primary messenger molecule. In contrast, other transmitters, i.e., acetylcholine, norepinephrine, dopamine, histamine and serotonin represent a small percentage of neurotransmission. The latest studies have demonstrated that an interaction between neurons and astrocytes is mediated by glutamatergic neurotransmissions [63]. Although glutamate concentration inside the synaptic cleft is elevated (ca. 100 mM), the basic concentration in the extracellular space is relatively low (ca. 2–40 µM) [64,65]. Glutamatergic neurotransmission is involved in normal brain function including cognition, memory and learning processes and in several neurological disorders such as schizophrenia, Parkinson’s disease, epilepsy, stroke and amyotrophic lateral sclerosis [61,66,67]. 

A glutamate biosensor was constructed based on a vertically aligned nickel nanowire array (NiNAE) and a Pt-coated nickel nanowire array (Pt/NiNAE) [68]. Both NiNAE and Pt/NiNAE electrodes demonstrated remarkably enhanced electrocatalytic activity towards glutamate compared to planar Ni electrodes, and demonstrated higher catalytic activity when compared to another metallic nanostructure electrodes such as a Pt-coated gold nanowire array electrode (Pt/AuNAE) and gold nanowire array electrodes (AuNAE).

### 3.2. Biogenic Amines Neurotransmitters

Biogenic amines are biologically important molecules that, like amino acids, have a nitrogen-containing amine group, but lack of carboxyl groups. The neurotransmitters based on biogenic amine are simple molecules that play critical roles in the regulation of central and peripheral nervous systems.

#### 3.2.1. Dopamine

Dopamine (DA) is one of the neurotransmitters, which plays an important role in the hormonal, cardiovascular, renal, and mammalian central nervous systems. Moreover, neurological disorders, such as Alzheimer’s and schizophrenia diseases incriminate abnormal levels of DA [69].

**Figure 1:**
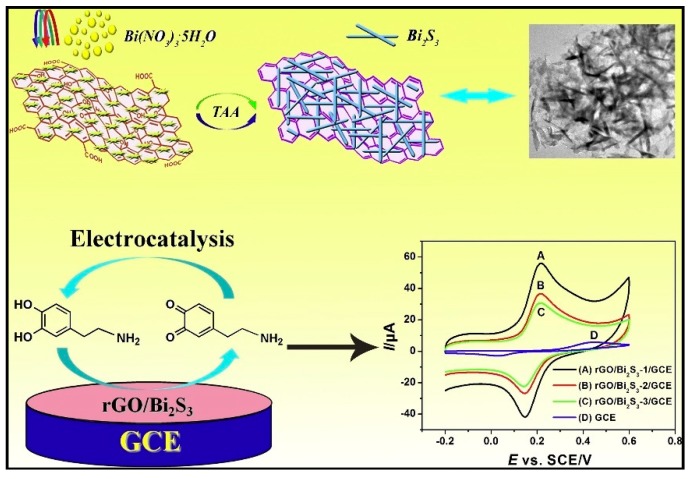
The schematic illustration of in-situ synthetic route of rGO/Bi_2_S_3_ composites and the electrocatalysis of DA at rGO/Bi_2_S_3_/GCE. Reprinted from [70] with permission of Elsevier.

Yan et al. developed a method for detection of DA in urine samples by Bi_2_S_3_ nanorods anchored over reduced graphene oxide (rGO/Bi_2_S_3_) [70]. rGO/Bi_2_S_3_ nanocomposite is synthesized by using thioacetamide as both reducing agent and sulfur source. Synthesis of rGO/Bi_2_S_3_ nanocomposites with tunable size by adjusting the dosage of GO was achieved. Also, rGO/Bi_2_S_3_ composite can accelerate electron transport and expand electrocatalytic active sites due to the unique construction and the synergistic effect between rGO and Bi_2_S_3_ nanorods, leading to the remarkable and stable current response for DA detection. By this method, DA was detected in a wide linear range of 0.01–40 µM and a low LOD of 12.3 nM was obtained (Figure 1).

The same group proposed a biomimetic sensor for the detection DA in real samples by introduction of electron-withdrawing groups into porphyrin molecules used as a cytochrome P450 model that can tune the energy level and have an effect on the electronic structure [71]. Linking with the strong electron withdrawing fluorine atoms, a starburst dendritic molecule, 5,10,15,20-tetrakis (pentafluorophenyl)−*21H*,23*H*-porphyrin iron (III) chloride (FeTFPP), containing a saddle-shaped porphyrin as the central core and four pentafluorophenyl rings as the peripheral functional groups was favorably synthesized. Afterward, the macrocyclic conjugate polymer film of FeTFPP was achieved via a low-cost electrochemical method and used as an efficient mimetic enzyme. A biomimetic sensor was constructed by the poly (FeTFPP) film and graphene (rGO) sheet (rGO-poly(FeTFPP)) for detection of DA.

**Figure 2:**
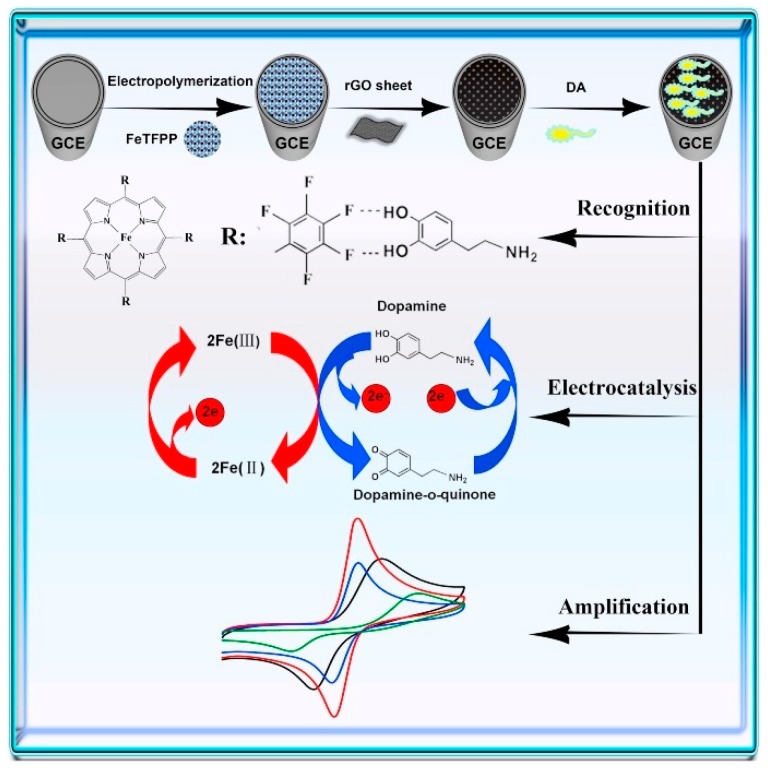
Scheme fabrication process of the biomimetic sensor and the catalytic process for the oxidation of dopamine. Reprinted from [71] with permission of Elsevier.

The DA response in the concentration range between 0.05 to 300 µΜ was linear with a LOD of 0.023 µΜ. The biomimetic sensor was used in real samples (urine and lake water) and satisfactory results were obtained, therefore, rGO-poly(FeTFPP) film represents a promising biomimetic catalyst for electrocatalysis and related fields (Figure 2) [71]. Another biomimetic electrochemical sensor for DA was developed based on AuNPs/GCE and electropolymerization of thioaniline in the presence of DA [94].

Different graphene-based electrochemical sensors were reported for DA detection. Firstly, the combination of rGO with polyurethane led to formation of a 3D porous material [72]. Physiological levels of DA were determined by an electrochemical sensor using the enhanced sensing features of processable graphene (pGr) obtained by mechanical ball milling of graphite. Higher electrocatalytic activity and lower LOD values were obtained for DA and ascorbic acid (AA) when compared with rGO. Prior the use, both rGO or pGr were first dispersed in DMF, then dropped over the pre-treated GCE carefully and allowed to dry for 24 h at room temperature [73].

Next, the electrochemical measurement of DA has been reported by a sensitive method with the aid of nanocomposite consist of rGO/zeolitic imidazolate framework-8 (rGO/ ZIF-8) [75].

As urine is one of the most common biological matrices for DA sensing, interferences must be considered. AA, uric acid (UA), amino acids and electrolytes are likely to be found in high concentrations in real samples. Therefore, besides the increased sensitivities resulting from different approaches, selectivity for DA sensing must be considered.

A water-based homogenous carbon ink-modified electrode has been used as an efficient sensor system for DA detection in the presence of AA and UA. A glassy carbon electrode was modified by carbon black and Chit. The CB-Chit ink-modified GCE (GCE/CB-Chit) displayed enhanced electrical conductivity, surface area and electrochemical activity compared to that of the unmodified GCE [86].

Another highly electrocatalytic nanocomposite for DA detection is represented by nickel tetrasulfonated phthalocyanine (NiTsPc) functionalized nitrogen-doped graphene (N-G) nanocomposites. The sensor probe was prepared by immobilization of NiTsPc on N-G matrix via *π-π* interactions. N-G provided a compatible microenvironment for NiTsPc to enhance electron transfer and to retain its electrocatalytic activity as well [77]. A similar approach for DA detection involves the use of nitrogen-doped graphene aerogels with 3D network structures fabricated using a hydrothermal method which includes the reduction of GO by an organic amine and self-assembly of rGO [35].

A hierarchical nanoporous (HNP) AuAg alloy was successfully prepared by a two-step dealloying process combined with an annealing operation [76]. HNP-AuAg composite displayed high electrocatalytic activity towards DA and UA in different potential regions. Hence, HNP-AuAg also showed good anti-interference against AA during the DA and UA detection.

DA detection was performed in the presence of UA using an electrochemical biosensor based on Au–SiO_2_ nanocomposite. A 215 mV peak to peak separation was obtained due to the higher electrocatalytic response of Au-SiO_2_/GCE when compared with Au/GCE and SiO_2_/GCE which is attributed to the increased surface area and conductivity of the nanocomposite. Differential pulse voltammetry measurements enabled the determination of two linear ranges from 10–100 μM and 200–500 μM with a LOD of 1.98 μM for DA and a linear range from 10–500 μM with a LOD of 2.58 μM for UA. Further, the detection of DA and UA in serum sample analysis was performed successfully with satisfactory recovery values [95].

A Cu-based metal−organic frameworks (Cu-MOFs) composite material was prepared and used to develop a sensitive and efficient electrochemical sensor for simultaneous detection of DA and paracetamol. AuNPs and the conductive poly(xanthurenic acid) p(XA) were assembled on the Cu-tetrakis (4-carboxyphenyl) porphyrin (TCPP) surface by electrodeposition and CV method, which greatly improved the electrocatalytic performance of the Cu-MOF modified electrode [96].

A 5 nm thickness poly-celestine blue (CB) modified GCE film showing excellent electrocatalytic activity toward the oxidation of DA was prepared by controlled electropolymerization. Two linear ranges were obtained by DPV measurements from 10 nM to 0.7 μM and 1 to 10 μM with a LOD of 1.2nM (S/N=3) and sensitivity of 17.01 μA cm^2^ μM^−1^. In addition, the poly-CB modified GCE has been successfully applied to determine nicotine-induced DA released from PC12 cells with satisfactory recovery [97].

#### 3.2.2. Epinephrine

Epinephrine (EP), also called adrenaline, is an excitatory neurotransmitter produced by the adrenal glands and released into the bloodstream. It prepares the body for the fight or flight reaction. It is one of the important catecholamine neurotransmitters that plays vital roles in the health of humans and other mammalians [98]. Many neurological, psychiatric and cardiovascular diseases are related to changes in biological EP concentrations. Therefore, it is necessary to develop quantitative methods for this catecholamine for studies on physiological function and diagnosis in clinical medicine [99].

Electropolymerization of ferulic acid (FA) at MWCNTs modified GCEs as a versatile platform for NADH enabled the determination of DA and EP. Chronoamperometry and cyclic voltammetry were employed to investigate the electrocatalytic oxidation of EP and DA at the modified electrode, in aqueous solutions. The obtained analytical curves for EP and DA showed linear ranges between 73–1406 μM, and 5–120 μM, respectively. The detection limits were 22 μM and 2 μM for EP and DA, respectively [78].

By contrast, Zhao et al. described the simultaneous measurement of EP and DA in body fluid samples by voltammetric methods using a modified GCE with dihexadecylphosphate film containing NiONPs and CNT. LODs of 82 nM and 50 nM in the linear range from 0.3 to 9.5 µM and from 70 nM to 4.8 µM were obtained for EP and DA, respectively [79].

Next, an electrochemical sensor using a modified carbon paste electrode (CPE) was fabricated for the simultaneous determination of EP in the presence of other two important interferents: DA and acetylcholine (ACh). The modifier used in this method was GO and 2-(5-ethyl-2,4-dihydroxyphenyl)-5,7-dimethyl-4*H*-pyrido[2,3-d] [1,3] thiazine-4-one (EDDPT). The detection limit and linear range of EP were 0.65 µM and 1.5–600.0 µM, respectively [88].

#### 3.2.3. Norepinephrine

Norepinephrine (NE) is a critical catecholamine neurotransmitter in the mammalian central nervous system. It is an endogenous hormone extruded by the adrenal medulla, and as a metabotropic neurotransmitter from nerve endings in the sympathetic nervous system and some spaces of the cerebral cortex. Low levels of NE cause depressive disorders. NE is also a major transmitter in many parts of the central nervous system, where it is involved in emotional arousal, blood pressure regulation, and mood disorders [100].

A NE sensor was constructed with a modified indium tin oxide (ITO) electrode with AuNPs. A stable layer of AuNPs was deposited on the surface of ITO showing increased electrocatalytic activity and a more positive peak potential towards NE. A LOD of 87 nM was calculated and linear range from 100 nM to 25 µM was obtained by (SWV) measurements. The sensor was applied for NE detection in biological fluids [89].

NTs-secreted from neuronal cells is the key role in regulating neural mechanism and various brain functions. Nowadays approaches are reporting in vitro monitoring of NTs-secreted from PC12 under K^+^ stimulation based on the highly advanced electrochemical sensor. Moreover, latest approaches reveal the multidetection of several neurotransmitters in complex matrices.

One of these nanocomposites with outstanding analytical performance is represented by NiO-lacy flower-like (NLF) geometrical structure with semi-spherical head surfaces connected with a trunk as an arm. The sensor was fabricated and employed for electrochemical screening of EP, NE, and DA released from dopaminergic cells. The novel structure of NLF shows the high surface area of 60.2 m^2^ g^−1^ with dominant mesoporous structure, and smooth surface of nanoneedles. Sensitive monitoring of NE, EP, and DA at low concentrations at the NLF-modified electrode was investigated by a chronoamperometric technique in 0.1 M PBS (pH = 7) at different applied potential for each target molecule (0.12 V-NE, 0.17 V-EP, 0.21 V-DA). LODs of 6 nM, 7 nM, and 8 nM were obtained for NE, EP and DA, respectively. The fabricated electrochemical electrode was successfully applied for detection of DA released from PC12-induced K^+^ in biological samples [101].

#### 3.2.4. Oxytocin

Oxytocin (Oxt) is a nonapeptide with many significant biological functions. Oxytocin has been attributed a role in social behavior in both male and female mammals. Oxytocin’s peripheral effects include the extraction of parturition and milk let-down, while central Oxt has been associated with the onset of maternal behavior and other positive social behaviors [102,103,104]. Asai et al. presented a method for electrochemical detection of Oxt using boron-doped diamond (BDD) microelectrodes over the concentration range from 0.1 to 10.0 μM with a detection limit of 50 nM (S/N = 3). By using this chronoamperometric method combined with flow injection analysis at an optimized potential, it was demonstrated that in situ or in vivo Oxt levels were selectively determined [90].

#### 3.2.5. Serotonin

Serotonin (5-hydroxytryptamine, 5-HT) is a redox-active monoamine neurotransmitter, often dispersed over all the central nervous system, which plays a fundamental task in various physiological processes, such as appetite, depression, liver regeneration, thermoregulation and in the regulation of mood, sleep, etc.

Sadanandhan et al. applied a new method to determine 5-HT using a PEDOTNTs/rGO/AgNPs hybrid nanocomposite-modified electrode as a transducer. The composition of nanostructured conducting polymers with rGO led to an improved electrocatalytic platform due to the combination of two excellent sensing materials. Moreover, by modifying the electrode surface with PEDOTNTs/rGO/AgNPs, a high increase in the charge transfer value (198 Ω) and good sensitivity towards the oxidation of 5-HT were obtained. Serotonin was measured in the linear range from 1 nM to 0.5 mM with a LOD of 0.1 nM in real samples. PEDOTNTs/rGO/AgNPs/GCE displayed excellent selectivity in the oxidation of serotonin in presence of AA, UA, Tyr that suggested its applicability in the selective detection of serotonin [91].

**Figure 3:**
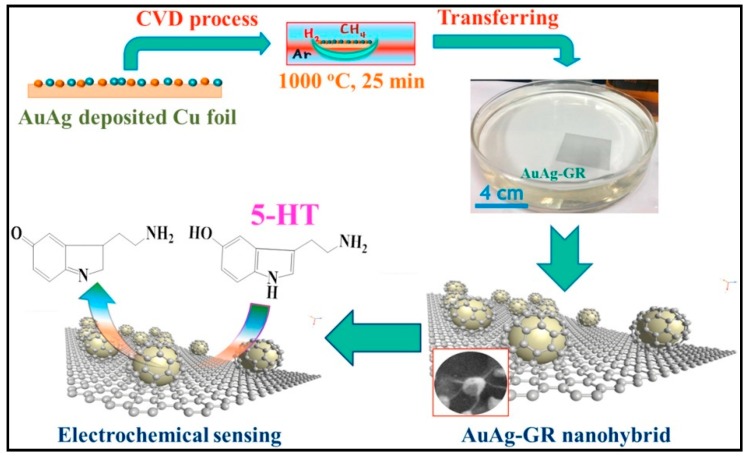
Scheme of fabrication and application of the AuAg-GR nanohybrid material for 5-HT detection. Reprinted from [92] with permission of Elsevier.

Furthermore, Tanha at el. developed a novel sensitive sensor for 5-HT based on high quality of graphene-encapsulated AuAg alloy (AuAg-GR) nanohybrid with homogeneous structure and good reproducibility (Figure 3). This method shows a dynamic linear range of serotonin from 2.7 nM to 4.82 μM with a very low detection limit (1.6 nM). In addition, the sensor was applied for 5-HT determination in human serum samples obtaining high recovery factors [92].

Another method reporting the highly selective and sensitive electrochemical detection of 5-HT is based on a customized platform by electrochemically-generated polypyrrole NPs (PPyNPs) decorated with AuNPs. A LOD of 33.22 nM and a sensitivity of 0.3316 μA μM^−1^ were obtained. The sensitivity towards 5-HT increased 320 fold after modification with the Au@PPy nanocomposite compared with bare electrode. The optimized platform was tested for DA and NE, lower sensitivities being observed for these compounds [105].

## 4. Enzyme Sensors

The electrochemical detection of non-electroactive species requires conversion into an electroactive analyte. Enzyme-based sensors undergo specific reactions that produce an electrochemically detectable signal, which can be useful for a plethora of non-electroactive analytes. Typically, they are highly selective and lower detection limits can be achieved.

Neurotransmitter detection has been successfully carried out in biological samples such as blood, serum, urine, and cerebrospinal liquid with the aid of electrochemical enzyme-based sensors and biosensors. Furthermore, it has been proved that some preliminary neurochemical tests could be performed in saliva samples, these biosensors’ representing a possible tool for population screening and for identifying underdiagnosed subjects in the very early stages of neurodisease development [106].

**Figure 4:**
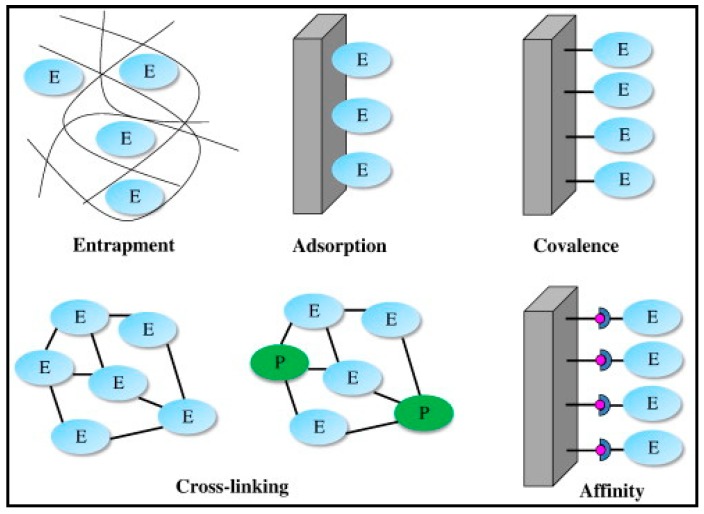
Schematic representation of main different methods for enzyme immobilization. Reprinted from [109] with permission of Elsevier.

An important property in designing the biorecognition part of enzymatic biosensors is the immobilization of the enzymes. Numerous books and comprehensive reviews have been written about enzyme immobilization [107,108,109,110]. There are various immobilization strategies such as: adsorption, covalence, entrapment, cross-linking or affinity (Figure 4) [109]. The best method of enzyme immobilization depends on different factors such as, the application, sensitivity, stability, and desired reproducibility [111,112,113].

The difficulty of the immobilization process also needs to be considered. The sensor sensitivity could be reduced if the immobilization process causes enzyme denaturation or conformational changes, or if the enzyme has been modified, especially in its active site. Therefore, enzymes should maintain their biological activity after immobilization, to remain firmly bound to the surface and not to be desorbed during their use.

Immobilization by entrapment is easy to perform. Enzymes, intermediates and additives can be simultaneously entrapped in the same sensing layer. There is no modification of the biological element therefore the activity of the enzyme is preserved during the immobilization process. Biosensors based on physically entrapped enzymes are often described to have increased operational and storage stability. However, conditions such as bioreceptor leaching and possible diffusion barriers can confine the performance of the systems. Moreover, an ideal biosensor has to be resistant for long-time application [109]. There are various approaches to immobilize enzymes by entrapment, such as the electropolymerization [114,115], amphiphilic networks [116], photopolymerization [117,118], sol–gel processes [119,120], polysaccharide-based gels [121], carbon pastes [122] and clay-modified electrodes [123,124]. Often, oxidoreductases, polyphenol oxidases, peroxidases, and amino oxidases are immobilized by entrapment procedures [125,126,127].

Several methods have also been reported for enzyme immobilization by using different nanomaterials [128,129,130,131,132,133]. Functionalized nanomaterials promote interactions between the electrode surface and the enzyme active center by means of increased electroactive surface area, improving sensitivity and electron transfer kinetics.

For sensing application, magnetic nanoparticles (MNPs) are used through direct application of tagged supports to the sensor, being integrated into the transducer materials, and/or dispersed in the sample followed by their attraction by an external magnetic field onto the active detection surface of the (bio)sensor [134]. This brings to the electrochemical biosensors an enhanced active surface improving the LOD and sensitivity as well as its easy reusability. A list of the most relevant enzymatic biosensors for neurological biomarkers analysis is summarized in Table 3.

### 4.1. Amino Acid Neurotransmitters

#### Glutamate

A glutamate biosensor was fabricated based on the electrocatalytic oxidation of reduced nicotinamide adenine dinucleotide (NADH) by employing thionine/single-walled carbon nanotubes (Th/SWNTs) nanocomposite as mediator and enzyme immobilization matrix. This biosensor exhibited a rapid response (5 s) in the linear range of 0.5–400 µM with a LOD of 0.1 µM [135]. By combining the highly performance of PPy/MWCNT nanocomposite at platinum (Pt) electrodes with the selective properties of glutamate oxidase (GlOx), glutamate detection was achieved by Ammam et al. Low response to interferences (AA, UA and paracetamol) was obtained due to the presence of a selective membrane of PPy and a LOD of 0.3 μM glutamate was calculated [136]. An amperometric microbiosensor was developed by using GlOx that entrapped in a biocompatible gel layer [137]. High sensitivity, fast response time, favorable selectivity and excellent stability were obtained, making the biosensor suitable for real time monitoring of l-glutamate released both in vitro and in vivo.

MWCNT and biopolymer Chit nanocomposite were used for the encapsulation of glutamate dehydrogenase (GlDH) and the co-factor nicotinamide adenine dinucleotide (NAD^+^) which were further deposited at the Meldola’s Blue (Mel B)-modified screen printed carbon electrode. As the base transducer involves a reagentless glutamate biosensor, a linear response was obtained in the range of 7.5–105 µM with a LOD of 3 µM and a sensitivity of 0.39 ± 0.025 nA µM^−1^ (RSD 6.37%, *n* = 5). The response time of the biosensor was 20–30 s [138].

Another material of interest is represented by titania and ceria nanoparticles, which were uniformly dispersed within a semipermeable chitosan membrane to develop a glutamate biosensor. This nanocomposite was co-immobilized with the enzyme GlOx at the surface of Pt microelectrodes. Amperometric measurements were performed at fixed potential (0.6V vs. Ag/AgCl) and a LOD of 0.5 μM and a sensitivity of 395 pA μM^−1^ (RSD 2.48%, *n* = 5) were obtained under oxygen-free conditions (5 s response time). Carbon-based materials and polymeric films have been increasingly used in biosensors’ development in the last decades. Moreover, by entrapping an enzyme in the electrode configuration leads to nanocomposites with enhanced electrochemical performance in terms of sensitivity and most important selectivity. Therefore, several enzymatic biosensors based on the use of carbon nanotubes/polymer hybrid composites are presented in the following subsections.

**Figure 5:**
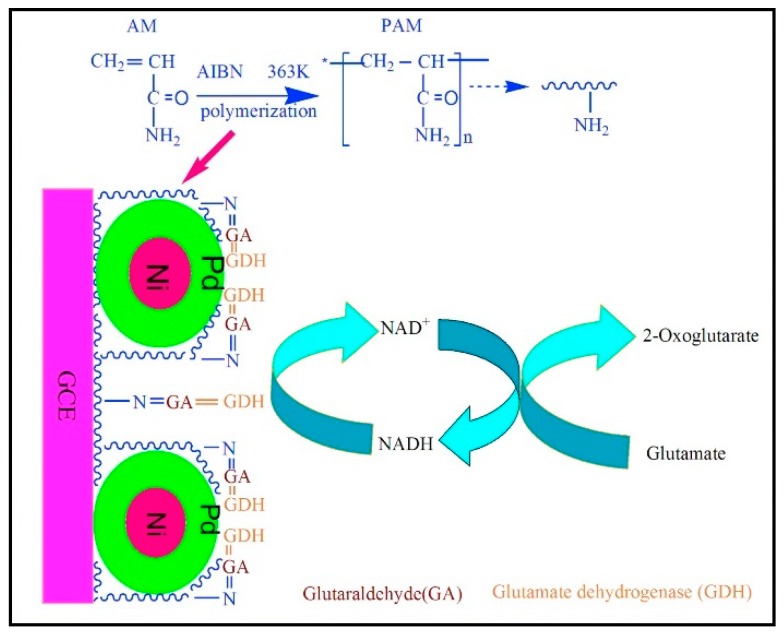
GlDH/NiePdePAM/GCE platform architecture. Reprinted from [156] with permission of Elsevier.

Preliminary in vivo glutamate monitoring were recorded in the cortex of Sprague-Dawley rats during cerebral ischemia and reperfusion demonstrating a potential application of the biosensor in hypoxic conditions [148]. Glutamate was also determined by using a modified glassy carbon electrode. This sensitive sensor was constructed for glutamate detection using thermal polymerization of acrylamide (AM) to immobilize Ni-Pd/core-shell NPs. For immobilizing NPs was used from polyacrylamide (PAM) film as a matrix, whereas the synthesized Ni-Pd/core-shell NPs act as electrocatalysts (Figure 5) [156].

A novel amperometric glutamate biosensor was developed based on covalent immobilization of GlOx onto carboxylated MWCNTs, AuNPs and Chit composite film electrodeposited on the surface of an Au electrode by Pundir and Batra [155].

Salazar et al. reported a Prussian blue-modified carbon fiber electrode (CFE/PB) to be used in microbiosensors for the determination of glutamate instead of the classical Pt and Pt-Ir transducers. The use of a PB-modified CFE provided sensitive H_2_O_2_ detection at a low applied potential (0.0V vs. SCE) and prevented bio-fouling and interference from other electroactive compounds. High sensitivity for glutamate (nA µM^−1^ cm^−2^) was achieved in a good linear range (up to 150 µM) with excellent anti-interference properties and low detection limit [149].

The synergistic effect of ZnONR and PPy nanocomposite at pencil graphite electrode was demonstrated by Barta et al. versus glutamate. The enzymatic amperometric biosensor was optimized with respect to pH, temperature, substrate concentration and time reaction, obtaining a LOD of 0.18 nM in the linear range of 0.02–500 µM [150]. In another work, glutamate biosensor has been developed by the covalent immobilization of GlOx onto PPyNPs and polyaniline composite film (PPyNPs/PANI) electrodeposited onto Au electrode [157].

An electrochemical biosensor was described for rapid glutamate detection by an implantable micromachined multi-electrode array modified microprobe. By an electrochemical deposition method, an iridium oxide (IrOx) film deposited onto a designated microelectrode enabled incorporation of an IrOx reference electrode in the microprobe [147].

### 4.2. Biogenic Amine Neurotransmitters

#### 4.2.1. Dopamine

Roychoudhury et al. reported an enzymatic biosensor for the detection of DA based on a nickel oxide nanoparticles (NiNPs) and tyrosinase (Tyr) enzyme conjugate. By a sol–gel method and using an ionic surfactant (sodium dodecylsulphate), controlled-sized NiNPS were synthesized. Tyr enzyme molecules were adsorbed on the NiNPs surface and thereafter enzyme-coated NPs were deposited on ITO-coated flexible polyethylene terephthalate (PET) substrate by a solution casting method. The proposed sensor showed good sensitivity of 60.2 nA µM^−1^ over a wide linear range (2–100 μM) with a LOD of 1.04 µM proving its successful applicability for point-of-care applications [143].

Fritea et al. developed two enzymatic biosensors for DA detection combining the specificity of the Tyr enzyme with the enhanced sensitivity at the electrode surface given by the special properties of layer-by-layer deposited GO/β-cyclodextrin [159], and rGO/PPy/β-cyclodextrin composite electrodes [144]. Both architectures showed enhanced electroactive surface area and high performances for dopamine biosensing with sensitivities and LODs of 0.017 A M^−1^ cm^−2^ and 3.9 µM [159] and 0.012 A M^−1^ cm^−2^ and 27 nM [144], respectively.

In vivo monitoring of dopamine levels will be helpful for physicians, but the use of enzymatic biosensors displays a number of drawbacks which limit their use: their instability, degradation in enzymatic activities and complex immobilization protocols, especially on microelectrodes demanded for sensing the fast releases of dopamine.

#### 4.2.2. Acetylcholine

Acetylcholine (ACh) is a neurotransmitter that mediates the chemical transmission of neuronal signals at synapses in the central and peripheral nervous systems. The ACh signal performs various biological functions, such as regulating physiological levels of different neurotransmitters, opening of ligand-gated ion channels, and bursting mode of neuronal firing. In hydrolysis and diffusion at synapses, imbalance of ACh is associated with several neurological and physiological diseases, such as Alzheimer’s [160] and myasthenia gravis [161]. In addition, levels of ACh change in behavioral, learning, and sleep disorders [162].

An interesting microfluidic structured-dual electrode approach was developed by Akhtar et al. The sensor is based on a pair of screen-printed carbon electrodes to detect ACh, where one of the working electrodes were used for the enzyme reaction evolution (a), as the second one for ACh detection (b) [139]. Firstly, the electrode was coated with AuNPs, whereas the latter with a porous gold layer, followed by electropolymerization of 2,2,5,2-terthiophene-3-(*p*-benzoic acid) (pTTBA) at both electrodes. Then, acetylcholinesterase was covalently attached onto the reaction electrode (a), and hydrazine and choline oxidase were co-immobilized on the detection electrode (b). After the modifications of both electrode surfaces, they precisely faced each other to form a microfluidic channel structure, where H_2_O_2_ produced from the sequential enzymatic reactions was reduced by hydrazine to obtain the analytical signal which was analyzed by the detection electrode (b). The microfluidic sensor at the optimized experimental conditions represented a wide dynamic range from 0.7 nM to 1.5 mM with a LOD of 0.6 ± 0.1 nM. Human plasma analysis was performed using the as developed sensor for ACh detection [139].

**Figure 6:**
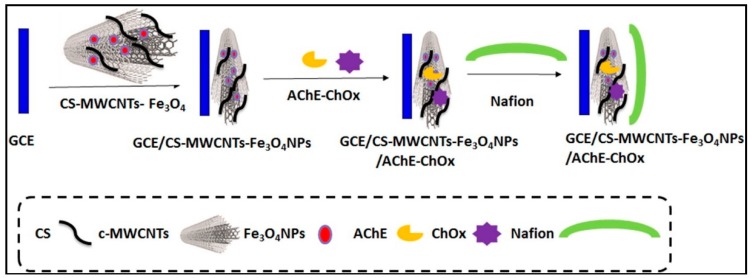
Schematic illustration of the stepwise ACh biosensor fabrication process. Reprinted from [141] with permission of Elsevier.

An amperometric bienzymatic biosensor for the determination of ACh was developed based on iron (II, III) oxide nanoparticles (Fe_3_O_4_NPs), and MWCNTs/chitosan modified GCE. Nafion membrane was used to fixing the modifier on the electrode surface. LOD of 0.61 nM and two linear ranges of 0.02–0.11 μM and 0.11–1.87 μM, respectively, were reported for this study (Figure 6) [141].

A self-powered amperometric biosensor for ACh detection in human plasma is described [140]. An effective immobilized acetylcholinesterase electrode was developed and its electrochemical performance evaluated by using highly porous gold as electrode material. The resulting enzymatic electrode was used as the anode of a miniature flow-through membrane-less fuel cell and indicated excellent response to varying concentrations of ACh. The peak power generated by the fuel cell at a voltage of 260 mV and with a current density of 9 μA cm^−2^ was 4 nW. The LOD of the fuel cell sensor was 10 μM in the linear range of 0.24–1.9 mM with an average response time as short as 3 min [140].

## 5. Biosensors for In Vitro, In Vivo and Ex Vivo Measurements

Monitoring variations in the NT concentration in biological fluids provides key information; however, their analyses, under in vitro, in vivo and ex vivo conditions is essential to offer a better understanding of the brain functioning and its disorders [4]. In this section, fundamental improvements resulting from recent studies by using biosensors have been highlighted.

During in vitro experiments the NTs released or secreted into extracellular liquid by the exocytosis process are detected. Some cell cultures (i.e., beige mouse mast cells, PC12 cells, human pancreatic beta cells, and chromaffin cells etc.) are used as non-synaptic in vitro models investigation achieving high sensitivity and suitable temporal resolution of secretory monoamine NTs [163].

Several other groups have investigated catecholamine release by monitoring exocytosis using different approaches [164,165,166,167,168,169]. The numerous studies carried out have gavin a great impulse to better understand the functions of the NTs but nevertheless they are not exhaustive. Therefore, in vivo studies are necessary to enhance the knowledge of the brain neurochemistry.

Non-invasive techniques such as positron emitting tomography (PET), magnetic resonance imaging (MRI) and magnetic resonance spectroscopy (MRS) are usually applied for in vivo monitoring of target analytes. However, these methods are limited by their low quantitative resolution and reduced temporal and/or spatial resolution [170]. Therefore, invasive methods such as implantable microbiosensors and microdialysis procedure are needed for additional in situ information.

The experimental protocol for implantable microbiosensors is as follows: first holes are drilled into the skull of the animal (weighed and anaesthetized) in the desired position; then, the release of NTs is caused electrically by placing bipolar electrodes in the brain areas; finally, the concentration of NTs is monitored [171,172,173,174].

Recently, implantable sensors with relatively fast response times and precise positioning are presented for glutamate [175] and for simultaneous detection of glutamate and DA by a nanocomposite-modified microelectrode array [176].

Ferreira et al. presented carbon fiber microelectrodes modified with Nafion^®^, CNTs, and ceramic-based microelectrode biosensor arrays for measuring ascorbate and glutamate in the brain with high spatial, temporal and chemical resolution [177].

Multiple cyclic SWV for analytical quantification of tonic dopamine concentrations in vivo with relatively high temporal resolution (10 s) was determined in the striatum of urethane anesthetized rats. DA concentration of 120 ± 18 nM (*n* = 7 rats, ± SEM) was obtained with high selectivity against AA, and 3,4-dihydroxyphenylacetic acid at different pHs [178].

In recent years, several research groups have been focusing on the application of on-line enzymatic biosensors for continuous screening of choline [179,180]. As the brain is a highly complex and sensitive part of body, detection of NT levels in the brain in the presence of a variety of interferences is a challenging task. In the last decades, numerous researchers have successfully coupled microdialysis with various biosensor platforms achieving good sensitivity for in vivo monitoring of NTs.

Microdialysis is the most commonly used sampling method for in vivo measurements of NTs from nerve endings to improve the sensitivity of the measurements. It is a very simple technique based on a tubular dialysis membrane inserted into a tissue or placed in contact with a wet surface, for example, a mucous membrane. The tube is perfused with a liquid that is put in equilibrium with the stream. According to this principle, the substances are able to pass from the point of view of the situation in which they are at a higher concentration, towards the one where it is less concentrated, until a dynamic equilibrium is reached in which two sides of the membrane. The degree of equilibrium is subject to the known laws of physical chemistry. The complexity of the technique derives from the complex interactions between the dialysis membrane, the perfusion fluid, and the surrounding tissue. The use of this technique is also correlated with the behavior of differences in extracellular concentrations of different NTs in the synaptic space. The coupling of the microdialysis with the analytical techniques (i.e., high performance liquid chromatography (HPLC) and capillary electrophoresis (CE)) is interesting enough to consider the endogenous substances of interest. Numerous researchers have successfully coupled microdialysis with various biosensors platforms.

Recently, monitoring DA concentration in brain microdialysate at 4 min intervals was successfully achieved by a sensor based on boronic acid-diol and *N*-hydroxysuccinimide ester-amine [181].

Zhang et al. [32] presented a novel biosensor which used a uniform dispersion of GO onto AuNPs to afford DA and 5-HT detection. The biosensor was inserted in electrolytic cell of micro-capacity connected with microdialysis platform. The LODs were 7.0 × 10^−9^ M for 5-HT and 5.6 × 10^−8^ M for DA.

However, microdialysis has significant limitations due to its relatively low temporal and spatial resolution [182]. In addition, despite its relative small dimensions, implantation of a biosensor or a microdialysis tube determines significant tissue damage and triggers foreign body reactions. There are a series of physiological processes that result in inactivation of the sensor, a process that is often referred to as “biofouling”. This process is one of the main reasons limiting their applicability. Therefore, ex vivo experiments are performed on brain slices providing quantitative data and benefits in comparison in the in vivo models.

The experimental protocol to initiate an experiment in an ex vivo model consists in quick removal of the brain from an animal and immediately keeping it in cold physiological saline solution. A tissue section is then cut and slices of the tissue are prepared. The technical advantages are due to the preclusion of the effects deriving from the use of anesthetics, the simplification of the neural network, the ability of a certain compound to cross the barrier blood-brain-barrier or the toxicity that a compound could have on the animal.

Over the last years, biosensors have been exploited for investigating monoamine NTs in various models [101]. A glutamate biosensor using GluOx was developed for analysis of rates of tonic, exocytotic and transporter-mediated glutamate release from isolated rat brain nerve terminals. Changes in the extracellular glutamate concentrations from 11.5 ± 0.9 to 11.7 ± 0.9 μΜ for 6min reflected a low tonic release of endogenous glutamate from nerve terminals [183].

Another interesting report on glutamate measurements in brain slices was based on a platinum microelectrode that demonstrated a fast response time fast (2 s) and low LOD of 44 nM [184].

A sensor for DA detection with excellent antifouling properties was presented by Zestos et al. [185]. The sensor was obtained by modification of CNT fiber electrode with polyethylenimine. The sensor was highly sensitive and selective detection of NTs with a LOD of 5 nM.

Other studies have been performed on the simultaneous measurement of NTs. A microarray has been realized by micro-electromechanical system technology for a rapid and sensitive determination in NE dynamic secretion. To improve the electrical performance, the MEA was electrodeposited with rGO and Pt nanoparticles. The extracellular NE secretion from the locus coeruleus brain slice, as well as monitoring spike firing from the hippocampal brain slice was successfully determined by the developed microarray [186].

## 6. Conclusions

In this review, the most recent developments in neurotransmitter detection based on electrochemical sensors with emphasis on enzymatic biosensors were presented. Electrochemical biosensors have many attractive analytical aspects and represent promising candidates for future clinical diagnostics due to their sensitive, simple, rapid and selective determination of NTs.

Research in nanocomposite-based biosensors has been growing exponentially in the last decade. Where past studies started with the use of one electrode modifier, typically derivatives of carbon nanomaterials, nowadays papers report hybrid compositions of numerous new types of 3D nanomaterials. By their integration in biosensor development outstanding analytical performance was achieved. Future challenges of several approaches remains for real samples analysis as robustness and reproducibility of the sensors are highly required.

Many of the electrochemical methods presented allow rapid, selective, and highly sensitive analysis of neurotransmitters in biological systems, without involving preparation steps; thus, they represent useful analytical tools that could be applied in clinical analysis. Using the current and future technology, another goal in electrochemical biosensing will be the development of implantable sensors for continuously monitoring human health states and disease development.

A major goal is represented by the simultaneous detection of a panel of neurotransmitters using electrode arrays and the real time and continuous monitoring. For this purpose, new nanomaterials and bioelements need to be integrated on the same platform in order to achieve long term stability, especially for implanted sensors exposed to biofouling. New techniques, like fast scan cycling voltammetry or scanning electrochemical microscopy should be developed for real time monitoring of NTs. Moreover, only a few materials are found to be biocompatible for in vivo applicationss; further studies are needed to explore and identify new biocompatible sensor platforms.

## Figures and Tables

**Table 1:** Classification of neurotransmitters, biological function and chemical structures [3].

Category	Neurotransmitter	Biological Function	Chemical Structure
Amino acid	Glutamate	cognition, memory and learning processes	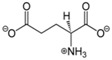
	Tyrosine	regulation of energy balance, memory, learning	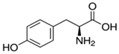
Biogenic amines	Dopamine	responsible for feelings of pleasure	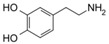
	Epinephrine	leading to a physical boost and heightened awareness	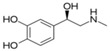
	Norepinephrine	improving attention and the speed at which responsive actions occur	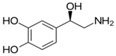
	Serotonin	regulating mood, sleep, emesis, sexuality, appetite, pain	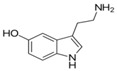
	Tryptamine	acting in central nervous system and gastrointestinal tract	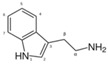
Acetyl choline	Acetylcholine	thought, learning and memory	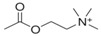
Soluble gases	Nitric oxide	cognitive functions, homeostatic functions, neurosecretion and synaptic plasticity	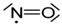
	Hydrogen sulphide	neuromodulator in the brain	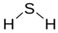

**Table 2:** Sensors for neurological biomarkers detections.

Neurological Biomarker	Electrode Surface	Linear Range; LOD (µM)	Sensitivity	Technique	Electrolyte	Real Samples/Storage	Interferences	Ref.
Dopamine	rGO/Bi_2_S_3_/GCE	0.01–40; 1.23 × 10^−2^	2.046 µA µM^−1^	CV–DPV	0.1 M PBS pH 6.0	Urine samples/30 days (91.6%)	Ca^2+^, Na^+^, Li^+^, Cu^2+^, Cl^−^, SO_4_^2−^, phenacetin, Glu, Fru, caffeine, APAP, Cys, Tyr, proline, AA, UA	[70]
	rGO-poly(FeTFPP)/GCE	0.05–300; 2.3 × 10^−2^	0.039 µA µM^−1^	CV–DPV-EIS	0.1 M PBS pH 6.0	Lake water; urine samples/long-term stability (90.2–93.6%)	Na^+^, Li^+^, Ca^2+^, Cl^−^, SO_4_^2−^, Glu, Mal, Fru, Lac, AA, UA	[71]
	rGO/PU	1 × 10^−4^–11.5 × 10^−4^; 1 × 10^−6^	0.011 µA pM^−1^	CV-DPV	0.05 M PBS pH 7.0	Human serum, urine/15 days (96.4%)	Fe^3+^, Zn^2+^, 4-NP, AA, UA, Tyr, Trp, GSH, Glu	[72]
	PGr/GCE	0.01–50; 1 × 10^−3^	0.478 µA µM^−1^	CV-DPV	0.1 M PBS pH 7.0	Human blood/30 days (89%)	AA, UA	[73]
		0.005–1; 1 × 10^−3^	0.004 µA µM^−1^				Simultaneous detection of DA and AA	
	rGO-Cu_2_O/GCE	10–900; 5 × 10^−2^	0.520 μA μM^−1^ cm^−2^	CV-DPV	0.1 M PBS pH 7.0	Human blood; urine/15 days (85%)	UA, AA, Glu, K^+^, Na^+^, Cl^−^, SO_4_^2−^	[74]
	rGO/ZIF-8/GCE	0.1–100; 3 × 10^−2^	0.153 μA μM^−1^	CV-DPV	0.1 M PBS pH 7.0	Human serum/15 days (95.7%)	AA	[75]
	HNP-AuAg/GCE	5–335; 2 × 10^−1^	0.399 μA μM^−1^ cm^−2^	CV-DPV-Amp	0.1 M PBS pH 7.0	-/ 20 days (99.3%)	AA / Simultaneous detection of DA and UA	[76]
	N-G/NiTsPC/GCE	0.1–200; 1 × 10^−1^	0.089 μA μM^−1^	Amp-CV-EIS	0.1 M PBS pH 7.4	- / 30 days (93.21%)	AA, UA	[77]
	poly-FA/MWCNT/GCE	5.00–120.0; 2.21	0.037 μA μM^−1^	Amp-CV	0.1 M PBS pH 7.0	Pharmaceutical samples/-	5-HT, AA, UA/Simultaneous detection of DA, NADH and EP	[78]
	NiO NP-MWCNT-DHP/GCE	0.07–4.8; 5 × 10^−2^	3.800 μA μM^−1^	DPV-SWV	0.2 M PBS pH 7.0	Cerebrospinal fluid, human serum and lung fluid/-	- / Simultaneous detection of DA, and EP	[79]
	HNP-PtTi/GCE	0.004–500; 3.2	0.186 μA μM^−1^ cm^−2^	CV-DPV-Amp	0.1 M PBS pH 7.0	Human serum/-	Na^+^, K^+^, Fe^3+^, Cu^2+^, Al^3+^, Glu, and H_2_O_2_ / Simultaneous detection of DA, UA and AA	[80]
	CPE/GO	0.08–2.30; 8.6 × 10^−3^	0.489 μA μM^−1^	CV-DPV-LSV	0.2 M Britton–Robinson buffer pH 4.0	Human blood/15 days (95.75%)	Na^+^, NH_4_^+^, NO_3_^−^, Cl^−^, CO_3_^2−^, K^+^, I^−^, phenylalanine, Cys, Trp/Simultaneous detection of DA in the presence of Tyr	[81]
	CE	0.4 – 100; 2 × 10^−1^	2.292 μA μM^−1^ cm^−2^	CV-DPV	0.1 M PBS pH 7.0	Human serum/14 days (95%)	Citric acid, Glu, Cys, l-glycine, Lys, Tyr, ANI, catechol, hydroquinone, phenol, resorcinol, Ca^2+^, K^+^, Mg^2+^, Na^+^, Zn^2+^/ Simultaneous detection of DA in the presence of AA, UA, Trp, and nitrite (NO_2_^−^)	[82]
	NiO-CuO/GR/GCE	0.5–20; 0.17	9.406 μA μM^−1^ cm^−2^	EIS-SWV	0.1 M PBS pH 8.0	Human serum, blood, pharmaceutical samples/30 days (95%)	K^+^, Na^+^, Zn^2+^, NO_3_^−^, Cl^−^, SO_4_^2−^/ Simultaneous detection of DA in the presence of APAP and Trp	[83]
	GR/p-AHNSA/SPCs	0.05–100; 2 × 10^−3^	0.099 μA μM^−1^	CV-EIS-SWV	0.1 M PBS pH 7.2	Human plasma, urine, pharmaceutical samples/-	AA, UA, Trp/Simultaneous detection of DA and 5-HT	[84]
	[AMIM][BF_4_]/CCE	0.1–20; 6.8 × 10^−2^	1.356 μA μM^−1^	CV-DPV	0.1 M PBS pH 7.0	Human blood serum, urine, pharmaceutical samples/20 days (96.6%)	Ca^2+^, Mg^2+^, Zn^2+^, Fe^3+^, K^+^, NO_2_^−^/ Simultaneous detection of DA and APAP	[85]
	CB-chit/GCE	0.1–1400; 1 × 10^−2^	0.132 μA μM^−1^	CV-DPV	0.1 M PBS pH 7.4	Human urine, pharmaceutical samples/-	-/ Simultaneous detection of DA and AA	[86]
Epinephrine	Paraffin/MWCNT/CoPc	1.33–5.50; 1.56 × 10^−2^	5.920 μA μM^−1^	DPV	0.2 M PBS pH 6.0	Human urine samples/(1000 determinations)	UA	[87]
	poly-FA/MWCNT/GCE	73.0–1406; 22.28	0.004 μA μM^−1^	Amp-DPV	0.1 M PBS pH 7.0	Pharmaceutical samples/-	5-HT, AA, UA/Simultaneous detection of DA, NADH and EP	[78]
	EDDPT/GO/CPE	1.5–600; 0.65	0.076 μA μM^−1^	DPV-Amp	0.1 M alkaline solution pH 7.0	Human serum, pharmaceutical samples/7 days (92%)	K^+^, Na^+^, Mg^2+^, Cl^−^, Glu, Fru, folic acid	[88]
	NiONP-MWCNT-DHP/GCE	0.3–9.5; 8.2 × 10^−2^	0.390 μA μM^−1^	DPV-SWV	0.2 M PBS pH 7.0	Cerebrospinal fluid, human serum and lung fluid/-	-/Simultaneous detection of DA, and EP	[79]
Glutamate	Pt/NiNAE	500–800; 83	0.096 μA μM^−1^ cm^−2^	Amp-CV	1 M NaOH	-/60 days (90%)	AA, UA, Glu	[68]
Norepinephrine	AuNPs/ITO	0.1–25; 8.7 × 10^−2^	1.011 μA μM^−1^	SWV-CV	0.1 M PBS pH 7.2	Human blood, urine/7 days (96.3%)	DA, UA, AA	[89]
Oxytocin	BDDE	1–10; 5 × 10^−2^	-	Amp-CV	0.1 M PBS pH 7.4	-	-/Selective detection of oxytocin and vasopresin	[90]
Serotonin	GR/p-AHNSA/SPCs	0.05–150; 3 × 10^−3^	0.101 μA μM^−1^	CV-EIS SWV	0.1 M PBS pH 7.2	Human plasma, urine, pharmaceutical samples/-	AA, UA, Trp/Simultaneous detection of DA and 5-HT	[84]
	PEDOTNTs/rGO/Ag NPs/GCE	0.01– 500; 1 × 10^−4^	0.0143 μA μM^−1^ cm^−2^	Amp-CV-DPV	0.1 M PBS pH 7.4	Bovine assayed multi-sera/30 days (97%)	Cys, Trp, Ala, Glu, DA, EP and NE/ Simultaneous detection of 5-HT in the presence of AA, UA, Tyr	[91]
	AuAgNPs/GR/ITO	0.0027–4.82 1.6 × 10^−3^	0.766 μA μM^−1^ cm^−2^	Amp-CV	0.1 M PBS pH 7.4	Human serum/19 days (88%)	Glu, K^+^, Cl-, UA, AA/-	[92]
Tryptamine	GCE	0.047–0.545 0.8 × 10^−3^	3.1 μA μM^−1^	SWADdSV	0.1 M Acetate buffer pH 5.3	Food samples/-	Putrescine/-	[93]

* 4-NP–4-nitrophenol; 5-HT–serotonin; AA–ascorbic acid; [AMIM][BF4]–1-allyl-3-methylimidazolium tetraflouroborate; Ala–alanine; Amp–chronoamperometry; ANI–aniline; APAP–acetaminophen; BBDE–boron-doped diamond electrode; CB–carbon black; Chit–chitosan; CE–carbon electrode; Cor–cortisol; CoPc–cobalt phthalocyanine; CPE–carbon paste electrode; CV–cyclic voltammetry; Cys–cysteine; DHP–dihexadecylphosphate; DPV–differential pulse voltammetry; EDDPT–2-(5-ethyl-2,4-dihydroxyphenyl)-5,7-dimethyl-4*H*-pyrido[2,3-d][1,3]thiazine-4-one; EIS–electrochemical impedance spectroscopy; FA–ferulic acid; FeTFPP–5,10,15,20-tetrakis(pentafluorophenyl)−21*H*,23*H*-porphyrin iron (III) chloride; Fru–fructose; GCE–glassy carbon electrode; GDE–graphite disk electrode; Glu–glucose; GO–graphene oxide; GR–graphene; GSH–glutathione, HNP–hierarchical nanoporous; Lac–lactose; LSV–linear sweep voltammetry; Lys–lysine; Mal–maltose; MrGO–magnetic functionalized reduced graphene oxide; MWCNTs–Multi walled carbon nanotubes; Naf–Nafion; NiNAE–—nickel nanowire array electrode; p-AHNSA–poly-4-amino-3-hydroxy-1-naphthalenesulfonic acid; PEDOT–poly(3,4 ethylenedioxythiophene; PPDpoly (phenylene diamine); PEDOTNTs–Poly(3,4-ethylenedioxythiophene) nanotubes; PU–Polyurethane; rGO–reduced graphene oxide; SPCs–screen-printed cells; SWADSV–square wave adsorptive stripping voltammetry; SWV–square wave voltammetry; Trp–Tryptophan; Tyr–tyrosine; VACNTs–vertically aligned carbon nanotubes; ZIF–8-zeolitic imidazolate.

**Table 3:** Enzymatic biosensors for neurological biomarkers detections.

Neurological Biomarker	Electrode Surface	Linear Range; LOD (µM)	Sensitivity	Technique	Electrolyte	Real Samples/Storage	Interferences	Ref.
Acetylcholine	SPCE/AuNPs/pTTBA-AChE	7 × 10^−4^ −60; 6 × 10^−4^	0.019 µA µM^−1^	Amp-CV-EIS	0.1 M PBS pH 7.4	Human plasma samples and cell line/60 days (91%)	AA, UA, catechol, GABA, APAP, DA, EP, glutamine	[139]
	AChE/hPG/ Pt	240–1900; 10	0.003 μA µM^−1^cm^−2^	CV-Amp-DPV-SWV	0.01 M Glycine pH 7.4 + 0.1 M of NaCl	-	-	[140]
	GCE/Chit-MWCNTs-Fe_3_O_4_NPs/AChE-ChOx	0.02–0.11; 6.1 × 10^−4^	5.890 µA µM^−1^	Amp-CV-EIS	0.05 M PBS pH 7.5	Human serum samples/30 days (60%)	AA, UA, APAP, Cys, Glu	[141]
Dopamine	HRP/MWCNTs	32–44; 2	1.980 µA µM^−1^	CV-DPV-SWV	0.25 M PBS pH 6.5	Pharmaceutical samples/Freezing 48 h (99.66%)	AA, UA	[142]
	Tyr/ NiONPs/ITO	2–200; 1.038	0.060 µA µM^−1^	CV	0.05M PBS pH 6.5	Fetal bovine serum samples/45 days (77%)	AA, UA	[143]
	rGO/β-CD-Py/GCE	0.027–38.6; 0.027	0.012 μA µM^−1^cm^−2^	Amp	0.1M PBS pH 6.5	-	AA, UA, Glu	[144]
Glutamate	GlDH-Th-SWCNTs/GCE	0.5–400; 0.1	0.137 μA µM^−1^ cm^−2^	CV-Amp	0.1 M PBS pH 8.3	-/14 days (93%)	AA, UA, APAP	[135]
	GlOx/ MWCNT/PPy/Pt	0.3–140; 0.3	0.384 μA µM^−1^ cm^−2^	Amp	0.1 M PBS pH 7.4	-/30 days (70%)	AA, UA, APAP	[136]
	GlOx/MWCNT/PAMAM/Pt/ Nafion	1.0–50.0; 0.5	0.002 μA µM^−1^	Amp-LSV	aCSF pH 7.4	Artificial cerebrospinal fluid/14 days (86%)	AA, DA/In vivo measurement of glutamate in the striatum of rats	[145]
	GlDH/VACNTs	0.1–20; 0.057	0.976 µA µM^−1^ cm^−2^	CV-DPV	0.1 M PBS pH 7.0	-/14 days (80.5%)	AA, UA	[146]
	GlOx/IrOx-MEA	5–300; 0.32	0.007 × 10^−3^ µA µM^−1^	Amp	PBS pH 7.2	-/14 days (71%)	AA, DA/In vitro and in vivo glutamate sensing	[147]
	CeO_2_/TiO_2_/GlOx/Chit/oPD/Pt	5–50; 0.493	0.793 × 10^−3^ µA µM^−1^	Amp	0.1 M PBS pH 7.4	Artificial cerebrospinal fluid/20 days (55%)	AA, DA, l-DOPA, 5-HT	[148]
	CFE/PoPD/GlOx/Gluth	0–150; 1.5	0.135 µA µM^−1^ cm^−2^	Amp	0.1 M PBS pH 7.4	-/30 days (90%)	Glu, lactate, 5-HT, glutamine, UA, AA	[149]
	GlOx/ZnONRs/PPy/PGE	0.02–500; 1.8 × 10^−4^	-	CV	0.1 M Tris–HCl pH 8.5	Food samples/90 days (70%)	-	[150]
	MWCNT-Chit-Mel B/GLDH-NAD^+^-Chit/MWCNT-Chit	7.5–105; 3.0	0.390 × 10^−3^ µA μM^−1^	CV-Amp	0.075 M PBS pH 7.0	Fetal bovine serum sample, food samples; / -	AA	[138]
	SHL-GlDH/oxygen electrode	10–1500; 3.0	0.087 × 10^−3^ µA μM^−1^	Amp	0.1 M Tris–HCl pH 8.0	- /14 days (≈100%)	AA, UA, 19 amino acids	[151]
	HBH-GlDH/oxygen electrode	10–1500; 5.0	0.089 × 10^−3^ µA μM^−1^	Amp	0.1 M PBS pH 6.5	- /7 days (70%)	AA, UA, 19 amino acids	[152]
	GlOx/PtNP/NAE	Up to 800; 14	0.011 μA μM^−1^ cm^−2^	Amp	0.01 M PBS pH 7.4	-/14 days (98%)	-	[153]
	GlDH-Chit-MelB/SPCE	12.5–150; 1.5	0.037 μA μM^−1^	Amp	0.075 M PBS pH 7.0	Fetal bovine serum sample, food samples; /-	-	[154]
	GlOx/cMWCNTs/AuNPs/Chit/ AuE	5–500; 1.6	0.155 μA μM^−1^ cm^−2^	CV-EIS	0.1 M PBS pH 7.5	Human serum samples;/4 months	AA, UA, Glu, bilirubin, urea, triglycerides	[155]
	GlDH/Ni-Pd-PAM/GCE	5–500; 0.052	4.768 μA μM^−1^ cm^−2^	CV-EIS-DPV	0.1 M PBS pH 7.4	Food samples; / 60 days (94.85%)	AA, Cys, l-aspartate	[156]
	GlOx-PPyNPs/PANI/AuE	0.02–400; 0.1 × 10^−3^	0.533 μA μM^−1^ cm^−2^	CV-EIS	0.1 M PBS pH 7.5	Food samples; / 60 days (70%)	AA, Glu, citric acid, Cys, methionine, lysine, aspartic acid, NaCl, glycine	[157]
	GlOx/PPD/Pt microelectrode	0.5–100 5 × 10^−3^	0.279 μA μM^−1^	Amp	0.1 M PBS pH 7.4	Artificial cerebrospinal fluid;/5 months (95%)	l-glutamine, l-aspartic acid, AA, DA, UA, 5-HT, catechol/In vivo glutamate sensing	[137]
Quinolinic acid	BSA/QPRT/rGO/ITO	6.5–65,000 6.5	7.860 × 10^3^ μA μM^−1^ cm^−2^	CV-DPV	PBS pH 7.0	Human serum samples;/30 days (95%)	-	[158]

* 5-HT—serotonin; AA—ascorbic acid; AChE—acetylcholinesterase; Amp—Chronoamperometry; APAP—acetaminophen; AuE—gold electrode; AuNPs—gold nanoparticles; BSA—bovine serum albumin; β-CD—β- cyclodextrin; CFE—carbon fiber electrode; Chit—chitosan; ChOx—choline oxidase; cMWCNTs—carboxylated multiwalled carbon nanotubes; Cys—cysteine; DPV—differential pulse voltammetry; EDC—1-ethyl-3-(3-dimethylaminopropyl) carbodiimide; EPI—Epinephrine; GABA—Gamma-Aminobutyric acid; GCE—glassy carbon electrode; GlOx—glutamate oxidase; GlDH—glutamate dehydrogenase; Glu—glucose; Gluth—glutaraldehyde; HBH—p-hydroxybenzoate hydroxylase; HRP—horseradish oeroxidase; hPG—highly porous gold; ITO—Indium Tin Oxide; l-DOPA—3,4-dihydroxy-l-phenylalanine; LSV—l1inear sweep voltammetry; MEA—micromachined multi-electrode array; MWCNT—Multiwalled carbon nanotubes; MelB—meldola’s blue; NAD^+^—nicotinamide adenine dinucleotide; NAEs—nanowire array electrodes; Opd— o- phenylenediamine; PAM—polyacrylamide; PAMAM—poly (amidoamine); PB—Prussian Blue; PEG—pyrolytic graphite electrode; PEI—polyethyleneimine; PPD—poly (phenylene diamine); Py—pyrrole; PPy—polypyrrole; PGE—pencil graphit electrode; PoPD—Poly ortho- phenylendiamine; PPyNPs—polypyrrole nanoparticles; PANI—polyaniline; PtNPs—platinum nanoparticles; pTTBA = 2, 2:5,2-terthiophene-3-(p-benzoic acid); PtNPs—Pt nanoparticles; QPRT—quinolinate phosphoribosyl transferase; rGO—reduced graphene oxide; SHL—salicylate hydroxylase; SPCE—screen-printed carbon electrode; SWCNTs—single walled carbon nanotyubes; Th—thionine; Tyr—Tyrosinase; UA—uric acid; VACNTs—vertically aligned carbon nanotubes; ZnO NRs—ZnO nanorods.

## References

[1] Costantino G. (1950). Elenco delle Cocciniglie osservate in Sicilia. Bolletino di Zool..

[2] Aoki I., Shirane K., Tokimoto T., Nakagawa K. (1986). Separation of fine particles using rotating tube with alternate flow. Rev. Sci. Instrum..

[3] Moon J.M., Thapliyal N., Hussain K.K., Goyal R.N., Shim Y.B. (2018). Conducting polymer-based electrochemical biosensors for neurotransmitters: A review. Biosens. Bioelectron..

[4] Baranwal A., Chandra P. (2018). Clinical implications and electrochemical biosensing of monoamine neurotransmitters in body fluids, in vitro, in vivo, and ex vivo models. Biosens. Bioelectron..

[5] Bucher E.S., Wightman R.M. (2015). Electrochemical Analysis of Neurotransmitters. Annu. Rev. Anal. Chem..

[6] Naveen M.H., Gurudatt N.G., Shim Y.B. (2017). Applications of conducting polymer composites to electrochemical sensors: A review. Appl. Mater. Today.

[7] Xia L., Wei Z., Wan M. (2010). Conducting polymer nanostructures and their application in biosensors. J. Colloid Interface Sci..

[8] Chauhan N., Chawla S., Pundir C.S., Jain U. (2017). An electrochemical sensor for detection of neurotransmitter-acetylcholine using metal nanoparticles, 2D material and conducting polymer modified electrode. Biosens. Bioelectron..

[9] Selvolini G., Băjan I., Hosu O., Cristea C., Săndulescu R., Marrazza G. (2018). DNA-based sensor for the detection of an organophosphorus pesticide: Profenofos. Sensors.

[10] Ravalli A., Rossi C., Marrazza G. (2017). Bio-inspired fish robot based on chemical sensors. Sens. Actuators B Chem..

[11] Rapini R., Cincinelli A., Marrazza G. (2016). Acetamiprid multidetection by disposable electrochemical DNA aptasensor. Talanta.

[12] Ferreira L.F., Souza L.M., Franco D.L., Castro A.C.H., Oliveira A.A., Boodts J.F.C., Brito-Madurro A.G., Madurro J.M. (2011). Formation of novel polymeric films derived from 4-hydroxybenzoic acid. Mater. Chem. Phys..

[13] Ferreira D.C., MacHado A.E.D.H., Tiago F.D.S., Madurro J.M., Madurro A.G.B., Abrahão O. (2012). Molecular modeling study on the possible polymers formed during the electropolymerization of 3-hydroxyphenylacetic acid. J. Mol. Graph. Model..

[14] Silva F.D.A.D.S., Lopes C.B., Kubota L.T., Lima P.R., Goulart M.O.F. (2012). Poly-xanthurenic acid modified electrodes: An amperometric sensor for the simultaneous determination of ascorbic and uric acids. Sens. Actuators B Chem..

[15] Song W., Chen Y., Xu J., Tian D.B. (2010). A selective voltammetric detection for dopamine using poly (gallic acid) film modified electrode. Chin. Chem. Lett..

[16] Herzog G., Gorgy K., Gulon T., Cosnier S. (2005). Electrogeneration and characterization of photoactivable films and their application for enzyme grafting. Electrochem. Commun..

[17] Hosu O., Elouarzaki K., Gorgy K., Cristea C., Sandulescu R., Marks R.S., Cosnier S. (2017). Nanostructured photoactivatable electrode surface based on pyrene diazirine. Electrochem. Commun..

[18] Hosu O., Bârsan M.M., Cristea C., Săndulescu R., Brett C.M.A. (2017). Nanostructured electropolymerized poly(methylene blue) films from deep eutectic solvents. Optimization and characterization. Electrochim. Acta.

[19] Peña R.C., Bertotti M., Brett C.M.A. (2011). Methylene Blue/Multiwall Carbon Nanotube Modified Electrode for the Amperometric Determination of Hydrogen Peroxide. Electroanalysis.

[20] Hosu O., Barsan M.M., Cristea C., Săndulescu R., Brett C.M.A. (2017). Nanocomposites based on carbon nanotubes and redox-active polymers synthesized in a deep eutectic solvent as a new electrochemical sensing platform. Microchim. Acta.

[21] Barsan M.M., Ghica M.E., Brett C.M.A. (2015). Electrochemical sensors and biosensors based on redox polymer/carbon nanotube modified electrodes: A review. Anal. Chim. Acta.

[22] Cui X., Lee V.A., Raphael Y., Wiler J.A., Hetke J.F., Anderson D.J., Martin D.C. (2001). Surface modification of neural recording electrodes with conducting polymer/biomolecule blends. J. Biomed. Mater. Res..

[23] Ku S., Palanisamy S., Chen S.M. (2013). Highly selective dopamine electrochemical sensor based on electrochemically pretreated graphite and nafion composite modified screen printed carbon electrode. J. Colloid Interface Sci..

[24] Zhao J., Yu Y., Weng B., Zhang W., Harris A.T., Minett A.I., Yue Z., Huang X.F., Chen J. (2013). Sensitive and selective dopamine determination in human serum with inkjet printed Nafion/MWCNT chips. Electrochem. Commun..

[25] Noroozifar M., Khorasani-Motlagh M., Hassani Nadiki H., Saeed Hadavi M., Mehdi Foroughi M. (2014). Modified fluorine-doped tin oxide electrode with inorganic ruthenium red dye-multiwalled carbon nanotubes for simultaneous determination of a dopamine, uric acid, and tryptophan. Sens. Actuators B Chem..

[26] Justino C.I.L., Rocha-Santos T.A.P., Cardoso S., Duarte A.C., Cardosa S. (2013). Strategies for enhancing the analytical performance of nanomaterial-based sensors. TrAC Trends Anal. Chem..

[27] Tertiş M., Hosu O., Fritea L., Farcau C., Cernat A., Săndulescu R., Cristea C. (2015). A Novel Label-Free Immunosensor Based on Activated Graphene Oxide for Acetaminophen Detection. Electroanalysis.

[28] Cernat A., Tertiş M., Săndulescu R., Bedioui F., Cristea A., Cristea C. (2015). Electrochemical sensors based on carbon nanomaterials for acetaminophen detection: A review. Anal. Chim. Acta.

[29] Atta N.F., El-Ads E.H., Ahmed Y.M., Galal A. (2016). Determination of some neurotransmitters at cyclodextrin/ionic liquid crystal/graphene composite electrode. Electrochim. Acta.

[30] Albishri H.M., Abd El-Hady D. (2019). Hyphenation of enzyme/graphene oxide-ionic liquid/glassy carbon biosensors with anodic differential pulse stripping voltammetry for reliable determination of choline and acetylcholine in human serum. Talanta.

[31] Wang Z., Ying Y., Li L., Xu T., Wu Y., Guo X., Wang F., Shen H., Wen Y., Yang H. (2017). Stretched graphene tented by polycaprolactone and polypyrrole net–bracket for neurotransmitter detection. Appl. Surf. Sci..

[32] Zhang S.J., Kang K., Niu L.M., Kang W.J. (2019). Electroanalysis of neurotransmitters via 3D gold nanoparticles and a graphene composite coupled with a microdialysis device. J. Electroanal. Chem..

[33] Ragavan K.V., Egan P., Neethirajan S. (2018). Multi mimetic Graphene Palladium nanocomposite based colorimetric paper sensor for the detection of neurotransmitters. Sens. Actuators B Chem..

[34] Tonel M.Z., González-Durruthy M., Zanella I., Fagan S.B. (2019). Interactions of graphene derivatives with glutamate-neurotransmitter: A parallel first principles—Docking investigation. J. Mol. Graph. Model..

[35] Ai S., Chen Y., Liu Y., Zhang Q., Xiong L., Huang H., Li L., Yu X., Wei L. (2018). Facile synthesis of nitrogen-doped graphene aerogels for electrochemical detection of dopamine. Solid State Sci..

[36] Raphey V.R., Henna T.K., Nivitha K.P., Mufeedha P., Sabu C., Pramod K. (2019). Advanced biomedical applications of carbon nanotube. Mater. Sci. Eng. C.

[37] Palanisamy S., Ku S., Chen S.M. (2013). Dopamine sensor based on a glassy carbon electrode modified with a reduced graphene oxide and palladium nanoparticles composite. Microchim. Acta.

[38] Niu L.M., Lian K.Q., Shi H.M., Wu Y.B., Kang W.J., Bi S.Y. (2013). Characterization of an ultrasensitive biosensor based on a nano-Au/DNA/nano-Au/poly(SFR) composite and its application in the simultaneous determination of dopamine, uric acid, guanine, and adenine. Sens. Actuators B Chem..

[39] Chávez J.L., Hagen J.A., Kelley-Loughnane N. (2017). Fast and selective plasmonic serotonin detection with Aptamer-gold nanoparticle conjugates. Sensors.

[40] Govindaraju S., Ankireddy S.R., Viswanath B., Kim J., Yun K. (2017). Fluorescent Gold Nanoclusters for Selective Detection of Dopamine in Cerebrospinal fluid. Sci. Rep..

[41] Kaur B., Srivastava R. (2015). A polyaniline-zeolite nanocomposite material based acetylcholinesterase biosensor for the sensitive detection of acetylcholine and organophosphates. New J. Chem..

[42] Ran G., Chen X., Xia Y. (2017). Electrochemical detection of serotonin based on a poly(bromocresol green) film and Fe3O4nanoparticles in a chitosan matrix. RSC Adv..

[43] Kergoat L., Piro B., Simon D.T., Pham M.C., Noël V., Berggren M. (2014). Detection of glutamate and acetylcholine with organic electrochemical transistors based on conducting polymer/platinum nanoparticle composites. Adv. Mater..

[44] Tsierkezos N.G., Ritter U., Nugraha Thaha Y., Knauer A., Fernandes D., Kelarakis A., McCarthy E.K. (2018). Boron-doped multi-walled carbon nanotubes as sensing material for analysis of dopamine and epinephrine in presence of uric acid. Chem. Phys. Lett..

[45] Si B., Song E. (2018). Recent Advances in the Detection of Neurotransmitters. Chemosensors.

[46] Ibáñez-Redín G., Wilson D., Gonçalves D., Oliveira O.N. (2018). Low-cost screen-printed electrodes based on electrochemically reduced graphene oxide-carbon black nanocomposites for dopamine, epinephrine and paracetamol detection. J. Colloid Interface Sci..

[47] Yang X., Feng B., He X., Li F., Ding Y., Fei J. (2013). Carbon nanomaterial based electrochemical sensors for biogenic amines. Microchim. Acta.

[48] Tąta A., Gralec B., Proniewicz E. (2018). Unsupported platinum nanoparticles as effective sensors of neurotransmitters and possible drug curriers. Appl. Surf. Sci..

[49] Wang C., Yuan R., Chai Y., Zhang Y., Hu F., Zhang M. (2011). Au-nanoclusters incorporated 3-amino-5-mercapto-1,2,4-triazole film modified electrode for the simultaneous determination of ascorbic acid, dopamine, uric acid and nitrite. Biosens. Bioelectron..

[50] Hanko M., Švorc Ľ., Planková A., Mikuš P. (2019). Overview and recent advances in electrochemical sensing of glutathione—A review. Anal. Chim. Acta.

[51] Shadlaghani A., Farzaneh M., Kinser D., Reid R.C. (2019). Direct Electrochemical Detection of Glutamate, Acetylcholine, Choline, and Adenosine Using Non-Enzymatic Electrodes. Sensors.

[52] Zhang Z., Wang X., Yang X. (2011). A sensitive choline biosensor using Fe_3_O_4_ magnetic nanoparticles as peroxidase mimics. Analyst.

[53] Fabregat G., Armelin E., Alemán C. (2014). Selective detection of dopamine combining multilayers of conducting polymers with gold nanoparticles. J. Phys. Chem. B.

[54] Rivnay J., Owens R.M., Malliaras G.G. (2014). The rise of organic bioelectronics. Chem. Mater..

[55] Yang Y.J., Li W. (2014). CTAB functionalized graphene oxide/multiwalled carbon nanotube composite modified electrode for the simultaneous determination of ascorbic acid, dopamine, uric acid and nitrite. Biosens. Bioelectron..

[56] Dakshayini B.S., Reddy K.R., Mishra A., Shetti N.P., Malode S.J., Basu S., Naveen S., Raghu A.V. (2019). Role of conducting polymer and metal oxide-based hybrids for applications in ampereometric sensors and biosensors. Microchem. J..

[57] Patrascu D., David I., David V., Mihailciuc C., Stamatin I., Ciurea J., Nagy L., Nagy G., Ciucu A.A. (2011). Selective voltammetric determination of electroactive neuromodulating species in biological samples using iron(II) phthalocyanine modified multi-wall carbon nanotubes paste electrode. Sens. Actuators B Chem..

[58] Chen X., Wu G., Cai Z., Oyama M., Chen X. (2014). Advances in enzyme-free electrochemical sensors for hydrogen peroxide, glucose, and uric acid. Microchim. Acta.

[59] Narayanan T.N., Vusa C.S.R., Alwarappan S. (2014). Selective and efficient electrochemical biosensing of ultrathin molybdenum disulfide sheets. Nanotechnology.

[60] Meldrum B.S. (2018). Glutamate as a Neurotransmitter in the Brain: Review of Physiology and Pathology. J. Nutr..

[61] Platt S.R. (2007). The role of glutamate in central nervous system health and disease—A review. Vet. J..

[62] Nedergaard M., Takano T., Hansen A.J. (2002). Beyond the role of glutamate as a neurotransmitter. Nat. Rev. Neurosci..

[63] Jessen R., Wood P.M., Wanner I.N.A.B., Mahoney J., An K., Bates M., Bunge M.B. (2006). Invariant Mantling of Growth Cones by Schwann Cell Precursors Characterize Growing Peripheral Nerve Fronts. Glia.

[64] Baurmash H.D. (2007). Transplantation of Alloplastic Submandibular Glands as Not Clinically Applicable Treatment for Xerostomia. J. Oral Maxillofac. Surg..

[65] Hinzman J.M., Thomas T.C., Quintero J.E., Gerhardt G.A., Lifshitz J. (2012). Disruptions in the Regulation of Extracellular Glutamate by Neurons and Glia in the Rat Striatum Two Days after Diffuse Brain Injury. J. Neurotrauma.

[66] Chang Y., Lin T.Y., Lu C.W., Huang S.K., Wang Y.C., Wang S.J. (2016). Xanthohumol-induced presynaptic reduction of glutamate release in the rat hippocampus. Food Funct..

[67] Wilson C.L., Natarajan V., Hayward S.L., Khalimonchuk O., Kidambi S. (2015). Mitochondrial dysfunction and loss of glutamate uptake in primary astrocytes exposed to titanium dioxide nanoparticles. Nanoscale.

[68] Jamal M., Hasan M., Mathewson A., Razeeb K.M. (2013). Disposable sensor based on enzyme-free Ni nanowire array electrode to detect glutamate. Biosens. Bioelectron..

[69] Diaz-Diestra D., Thapa B., Beltran-Huarac J., Weiner B.R., Morell G. (2017). l-cysteine capped ZnS:Mn quantum dots for room-temperature detection of dopamine with high sensitivity and selectivity. Biosens. Bioelectron..

[70] Yan X., Gu Y., Li C., Zheng B., Li Y., Zhang T., Zhang Z., Yang M. (2018). Morphology-controlled synthesis of Bi2S3 nanorods-reduced graphene oxide composites with high-performance for electrochemical detection of dopamine. Sens. Actuators B Chem..

[71] Yan X., Lu N., Gu Y., Li C., Zhang T., Liu H., Zhang Z., Zhai S. (2018). Catalytic activity of biomimetic model of cytochrome P450 in oxidation of dopamine. Talanta.

[72] Vilian A.T.E., An S., Choe S.R., Kwak C.H., Huh Y.S., Lee J., Han Y.K. (2016). Fabrication of 3D honeycomb-like porous polyurethane-functionalized reduced graphene oxide for detection of dopamine. Biosens. Bioelectron..

[73] Ramachandran A., Panda S., Karunakaran Yesodha S. (2018). Physiological level and selective electrochemical sensing of dopamine by a solution processable graphene and its enhanced sensing property in general. Sens. Actuators B Chem..

[74] Sivasubramanian R., Biji P. (2016). Preparation of copper (I) oxide nanohexagon decorated reduced graphene oxide nanocomposite and its application in electrochemical sensing of dopamine. Mater. Sci. Eng. B Solid-State Mater. Adv. Technol..

[75] Yu G., Xia J., Zhang F., Wang Z. (2017). Hierarchical and hybrid RGO/ZIF-8 nanocomposite as electrochemical sensor for ultrasensitive determination of dopamine. J. Electroanal. Chem..

[76] Hou J., Xu C., Zhao D., Zhou J. (2016). Facile fabrication of hierarchical nanoporous AuAg alloy and its highly sensitive detection towards dopamine and uric acid. Sens. Actuators B Chem..

[77] Xu H., Xiao J., Yan L., Zhu L., Liu B. (2016). An electrochemical sensor for selective detection of dopamine based on nickel tetrasulfonated phthalocyanine functionalized nitrogen-doped graphene nanocomposites. J. Electroanal. Chem..

[78] da Silva L.V., Lopes C.B., da Silva W.C., de Paiva Y.G., Silva F., de Assis dos Santos Silva F., Lima P.R., Kubota L.T., Goulart M.O.F. (2017). Electropolymerization of ferulic acid on multi-walled carbon nanotubes modified glassy carbon electrode as a versatile platform for NADH, dopamine and epinephrine separate detection. Microchem. J..

[79] Figueiredo-Filho L.C.S., Silva T.A., Vicentini F.C., Fatibello-Filho O. (2014). Simultaneous voltammetric determination of dopamine and epinephrine in human body fluid samples using a glassy carbon electrode modified with nickel oxide nanoparticles and carbon nanotubes within a dihexadecylphosphate film. Analyst.

[80] Zhao D., Yu G., Tian K., Xu C. (2016). A highly sensitive and stable electrochemical sensor for simultaneous detection towards ascorbic acid, dopamine, and uric acid based on the hierarchical nanoporous PtTi alloy. Biosens. Bioelectron..

[81] Ghoreishi S.M., Behpour M., Mortazavi M., Khoobi A. (2016). Fabrication of a graphene oxide nano-sheet modified electrode for determination of dopamine in the presence of tyrosine: A multivariate optimization strategy. J. Mol. Liq..

[82] Sun L., Li H., Li M., Li C., Li P., Yang B. (2016). Simultaneous determination of ascorbic acid, dopamine, uric acid, tryptophan, and nitrite on a novel carbon electrode. J. Electroanal. Chem..

[83] Liu B., Ouyang X., Ding Y., Luo L., Xu D., Ning Y. (2016). Electrochemical preparation of nickel and copper oxides-decorated graphene composite for simultaneous determination of dopamine, acetaminophen and tryptophan. Talanta.

[84] Raj M., Gupta P., Goyal R.N., Shim Y.B. (2017). Graphene/conducting polymer nano-composite loaded screen printed carbon sensor for simultaneous determination of dopamine and 5-hydroxytryptamine. Sens. Actuators B Chem..

[85] Majidi M.R., Pournaghi-Azar M.H., Fadakar Bajeh Baj R. (2016). Graphene nanoplatelets like structures formed on ionic liquid modified carbon-ceramic electrode: As a sensing platform for simultaneous determination of dopamine and acetaminophen. J. Mol. Liq..

[86] Dinesh B., Saraswathi R., Senthil Kumar A. (2017). Water based homogenous carbon ink modified electrode as an efficient sensor system for simultaneous detection of ascorbic acid, dopamine and uric acid. Electrochim. Acta.

[87] Moraes F.C., Golinelli D.L.C., Mascaro L.H., MacHado S.A.S. (2010). Determination of epinephrine in urine using multi-walled carbon nanotube modified with cobalt phthalocyanine in a paraffin composite electrode. Sens. Actuators B Chem..

[88] Tezerjani M.D., Benvidi A., Dehghani Firouzabadi A., Mazloum-Ardakani M., Akbari A. (2017). Epinephrine electrochemical sensor based on a carbon paste electrode modified with hydroquinone derivative and graphene oxide nano-sheets: Simultaneous determination of epinephrine, acetaminophen and dopamine. Meas. J. Int. Meas. Confed..

[89] Goyal R.N., Aziz M.A., Oyama M., Chatterjee S., Rana A.R.S. (2011). Nanogold based electrochemical sensor for determination of norepinephrine in biological fluids. Sens. Actuators B Chem..

[90] Asai K., Ivandini T.A., Einaga Y. (2016). Continuous and selective measurement of oxytocin and vasopressin using boron-doped diamond electrodes. Sci. Rep..

[91] Sadanandhan N.K., Cheriyathuchenaaramvalli M., Devaki S.J., Ravindranatha Menon A.R. (2017). PEDOT-reduced graphene oxide-silver hybrid nanocomposite modified transducer for the detection of serotonin. J. Electroanal. Chem..

[92] Thanh T.D., Balamurugan J., Van Hien H., Kim N.H., Lee J.H. (2017). A novel sensitive sensor for serotonin based on high-quality of AuAg nanoalloy encapsulated graphene electrocatalyst. Biosens. Bioelectron..

[93] Costa D.J.E., Martínez A.M., Ribeiro W.F., Bichinho K.M., Di Nezio M.S., Pistonesi M.F., Araujo M.C.U. (2016). Determination of tryptamine in foods using square wave adsorptive stripping voltammetry. Talanta.

[94] Tertiş M., Florea A., Adumitrăchioaie A., Cernat A., Bogdan D., Barbu-Tudoran L., Jaffrezic Renault N., Săndulescu R., Cristea C. (2017). Detection of Dopamine by a Biomimetic Electrochemical Sensor Based on Polythioaniline-Bridged Gold Nanoparticles. Chempluschem.

[95] Immanuel S., Aparna T.K., Sivasubramanian R. (2019). A facile preparation of Au—SiO2 nanocomposite for simultaneous electrochemical detection of dopamine and uric acid. Surf. Interfaces.

[96] Qiu Z., Yang T., Gao R., Jie G., Hou W. (2019). An electrochemical ratiometric sensor based on 2D MOF nanosheet/Au/polyxanthurenic acid composite for detection of dopamine. J. Electroanal. Chem..

[97] Yang C., Liu M.M., Bai F.Q., Guo Z.Z., Liu H., Zhong G.X., Peng H.P., Chen W., Lin X.H., Lei Y. (2019). An electrochemical biosensor for sensitive detection of nicotine-induced dopamine secreted by PC12 cells. J. Electroanal. Chem..

[98] Mazloum-Ardakani M., Beitollahi H., Mohseni M.A.S., Benvidi A., Naeimi H., Nejati-Barzoki M., Taghavinia N. (2010). Simultaneous determination of epinephrine and acetaminophen concentrations using a novel carbon paste electrode prepared with 2,2′-[1,2 butanediylbis(nitriloethylidyne)]-bis-hydroquinone and TiO2 nanoparticles. Colloids Surf. B Biointerfaces.

[99] Michael D.J., Wightman R.M. (1999). Electrochemical monitoring of biogenic amine neurotransmission in real time. J. Pharm. Biomed. Anal..

[100] Mazloum-Ardakani M., Beitollahi H., Amini M.K., Mirkhalaf F., Mirjalili B.F. (2011). A highly sensitive nanostructure-based electrochemical sensor for electrocatalytic determination of norepinephrine in the presence of acetaminophen and tryptophan. Biosens. Bioelectron..

[101] Emran M.Y., Shenashen M.A., Mekawy M., Azzam A.M., Akhtar N., Gomaa H., Selim M.M., Faheem A., El-Safty S.A. (2018). Ultrasensitive in-vitro monitoring of monoamine neurotransmitters from dopaminergic cells. Sens. Actuators B Chem..

[102] Kosfeld M., Heinrichs M., Zak P.J., Fischbacher U., Fehr E. (2005). Oxytocin increases trust in humans. Nature.

[103] Pedersen C.A., Boccia M.L. (2002). Oxytocin links mothering received, mothering bestowed and adult stress responses. Stress.

[104] Bales K.L., van Westerhuyzen J.A., Lewis-Reese A.D., Grotte N.D., Lanter J.A., Carter C.S. (2007). Oxytocin has dose-dependent developmental effects on pair-bonding and alloparental care in female prairie voles. Horm. Behav..

[105] Tertiș M., Cernat A., Lacatiș D., Florea A., Bogdan D., Suciu M., Săndulescu R., Cristea C. (2017). Highly selective electrochemical detection of serotonin on polypyrrole and gold nanoparticles-based 3D architecture. Electrochem. Commun..

[106] Gug I.T., Tertis M., Hosu O., Cristea C. (2019). Salivary biomarkers detection: Analytical and immunological methods overview. TrAC Trends Anal. Chem..

[107] Cao L. (2005). Carrier-Bound Immobilized Enzymes.

[108] Mateo C., Palomo J.M., Fernandez-Lorente G., Guisan J.M., Fernandez-Lafuente R. (2007). Improvement of enzyme activity, stability and selectivity via immobilization techniques. Enzyme Microb. Technol..

[109] Sassolas A., Blum L.J., Leca-Bouvier B.D. (2012). Immobilization strategies to develop enzymatic biosensors. Biotechnol. Adv..

[110] Brena B., González-Pombo P., Batista-Viera F., Guisan J. (2013). Immobilization of enzymes: A literature survey. Methods in Molecular Biology.

[111] Choi M.M.F. (2004). Progress in enzyme-based biosensors using optical transducers. Microchim. Acta.

[112] Arya S.K., Datta M., Malhotra B.D. (2008). Recent advances in cholesterol biosensor. Biosens. Bioelectron..

[113] Andreescu S., Marty J.L. (2006). Twenty years research in cholinesterase biosensors: From basic research to practical applications. Biomol. Eng..

[114] Sassolas A., Blum L.J., Leca-Bouvier B.D. (2009). New electrochemiluminescent biosensors combining polyluminol and an enzymatic matrix. Anal. Bioanal. Chem..

[115] Tembe S., Karve M., Inamdar S., Haram S., Melo J., D’Souza S.F. (2006). Development of electrochemical biosensor based on tyrosinase immobilized in composite biopolymeric film. Anal. Biochem..

[116] Hanko M., Bruns N., Tiller J.C., Heinze J. (2006). Optical biochemical sensor for determining hydroperoxides in nonpolar organic liquids as archetype for sensors consisting of amphiphilic conetworks as immobilisation matrices. Anal. Bioanal. Chem..

[117] Galezowska A., Sikora T., Istamboulie G., Trojanowicz M., Polec I., Nunes G.S., Noguer T., Marty J.L. (2017). Application of Genetically Engineered Acetylcholinesterases in Screen-Printed Amperometric Biosensor for Detection of Organophosphorus Insecticides. Sens. Mater..

[118] Valdés-Ramírez G., Cortina M., Ramírez-Silva M.T., Marty J.L. (2008). Acetylcholinesterase-based biosensors for quantification of carbofuran, carbaryl, methylparaoxon, and dichlorvos in 5% acetonitrile. Anal. Bioanal. Chem..

[119] Gupta R., Chaudhury N.K. (2007). Entrapment of biomolecules in sol-gel matrix for applications in biosensors: Problems and future prospects. Biosens. Bioelectron..

[120] Jerónimo P.C.A., Araújo A.N., Conceição M. (2007). Optical sensors and biosensors based on sol-gel films. Talanta.

[121] Prakash O., Talat M., Hasan S.H., Pandey R.K. (2008). Enzymatic detection of heavy metal ions in aqueous solution from vegetable wastes by immobilizing pumpkin (Cucumis melo) urease in calcium alginate beads. Biotechnol. Bioprocess Eng..

[122] Švancara I., Vytřas K., Kalcher K., Walcarius A., Wang J. (2009). Carbon paste electrodes in facts, numbers, and notes: A review on the occasion of the 50-years jubilee of carbon paste in electrochemistry and electroanalysis. Electroanalysis.

[123] An N., Zhou C.H., Zhuang X.Y., Tong D.S., Yu W.H. (2015). Immobilization of enzymes on clay minerals for biocatalysts and biosensors. Appl. Clay Sci..

[124] Mousty C. (2010). Biosensing applications of clay-modified electrodes: A review. Anal. Bioanal. Chem..

[125] Jaafar M.M., Ciniciato G.P.M.K., Ibrahim S.A., Phang S.M., Yunus K., Fisher A.C., Iwamoto M., Vengadesh P. (2015). Preparation of a Three-Dimensional Reduced Graphene Oxide Film by Using the Langmuir-Blodgett Method. Langmuir.

[126] Mehrotra P. (2016). Biosensors and their applications—A review. J. Oral Biol. Craniofacial Res..

[127] Akyilmaz E., Yorganci E., Asav E. (2010). Do copper ions activate tyrosinase enzyme? A biosensor model for the solution. Bioelectrochemistry.

[128] Fortes C.C.S., Daniel-da-Silva A.L., Xavier A.M.R.B., Tavares A.P.M. (2017). Optimization of enzyme immobilization on functionalized magnetic nanoparticles for laccase biocatalytic reactions. Chem. Eng. Process. Process Intensif..

[129] Cao S., Xu P., Ma Y., Yao X., Yao Y., Zong M., Li X., Lou W. (2016). Recent advances in immobilized enzymes on nanocarriers. Cuihua Xuebao/Chin. J. Catal..

[130] Khoshnevisan K., Vakhshiteh F., Barkhi M., Baharifar H., Poor-Akbar E., Zari N., Stamatis H., Bordbar A.K. (2017). Immobilization of cellulase enzyme onto magnetic nanoparticles: Applications and recent advances. Mol. Catal..

[131] Kim H.J., Park S., Kim S.H., Kim J.H., Yu H., Kim H.J., Yang Y.H., Kan E., Kim Y.H., Lee S.H. (2015). Biocompatible cellulose nanocrystals as supports to immobilize lipase. J. Mol. Catal. B Enzym..

[132] Deng X., Cao S., Li N., Wu H., Smith T.J., Zong M., Lou W. (2016). A magnetic biocatalyst based on mussel-inspired polydopamine and its acylation of dihydromyricetin. Cuihua Xuebao/Chin. J. Catal..

[133] Cao S., Huang Y., Li X., Xu P., Wu H., Li N., Lou W. (2016). Preparation and Characterization of Immobilized Lipase from Pseudomonas Cepacia onto Magnetic Cellulose Nanocrystals. Nat. Publ. Gr..

[134] Rocha-Santos T.A.P. (2014). Sensors and biosensors based on magnetic nanoparticles. TrAC Trends Anal. Chem..

[135] Meng L., Wu P., Chen G., Cai C., Sun Y., Yuan Z. (2009). Low potential detection of glutamate based on the electrocatalytic oxidation of NADH at thionine/single-walled carbon nanotubes composite modified electrode. Biosens. Bioelectron..

[136] Ammam M., Fransaer J. (2010). Highly sensitive and selective glutamate microbiosensor based on cast polyurethane/AC-electrophoresis deposited multiwalled carbon nanotubes and then glutamate oxidase/electrosynthesized polypyrrole/Pt electrode. Biosens. Bioelectron..

[137] Tian F., Gourine A.V., Huckstepp R.T.R., Dale N. (2009). A microelectrode biosensor for real time monitoring of l-glutamate release. Anal. Chim. Acta.

[138] Hughes G., Pemberton R.M., Fielden P.R., Hart J.P. (2015). Development of a novel reagentless, screen-printed amperometric biosensor based on glutamate dehydrogenase and NAD^+^, integrated with multi-walled carbon nanotubes for the determination of glutamate in food and clinical applications. Sens. Actuators B Chem..

[139] Akhtar M.H., Hussain K.K., Gurudatt N.G., Shim Y.B. (2017). Detection of Ca2+-induced acetylcholine released from leukemic T-cells using an amperometric microfluidic sensor. Biosens. Bioelectron..

[140] Moreira F.T.C., Sale M.G.F., Di Lorenzo M. (2017). Towards timely Alzheimer diagnosis: A self-powered amperometric biosensor for the neurotransmitter acetylcholine. Biosens. Bioelectron..

[141] Bolat E.Ö., Tığ G.A., Pekyardımcı Ş. (2017). Fabrication of an amperometric acetylcholine esterase-choline oxidase biosensor based on MWCNTs-Fe_3_O_4_NPs-CS nanocomposite for determination of acetylcholine. J. Electroanal. Chem..

[142] De Souza Ribeiro F.A., Tarley C.R.T., Borges K.B., Pereira A.C. (2013). Development of a square wave voltammetric method for dopamine determination using a biosensor based on multiwall carbon nanotubes paste and crude extract of *Cucurbita pepo* L.. Sens. Actuators B Chem..

[143] Roychoudhury A., Basu S., Jha S.K. (2016). Dopamine biosensor based on surface functionalized nanostructured nickel oxide platform. Biosens. Bioelectron..

[144] Fritea L., Le Goff A., Putaux J.L., Tertis M., Cristea C., Səndulescu R., Cosnier S. (2015). Design of a reduced-graphene-oxide composite electrode from an electropolymerizable graphene aqueous dispersion using a cyclodextrin-pyrrole monomer. Application to dopamine biosensing. Electrochim. Acta.

[145] Yu Y., Sun Q., Zhou T., Zhu M., Jin L., Shi G. (2011). On-line microdialysis system with poly(amidoamine)-encapsulated Pt nanoparticles biosensor for glutamate sensing in vivo. Bioelectrochemistry.

[146] Gholizadeh A., Shahrokhian S., Iraji zad A., Mohajerzadeh S., Vosoughi M., Darbari S., Sanaee Z. (2012). Mediator-less highly sensitive voltammetric detection of glutamate using glutamate dehydrogenase/vertically aligned CNTs grown on silicon substrate. Biosens. Bioelectron..

[147] Tolosa V.M., Wassum K.M., Maidment N.T., Monbouquette H.G. (2013). Electrochemically deposited iridium oxide reference electrode integrated with an electroenzymatic glutamate sensor on a multi-electrode array microprobe. Biosens. Bioelectron..

[148] Özel R.E., Ispas C., Ganesana M., Leiter J.C., Andreescu S. (2014). Glutamate oxidase biosensor based on mixed ceria and titania nanoparticles for the detection of glutamate in hypoxic environments. Biosens. Bioelectron..

[149] Salazar P., Martín M., O’Neill R.D., González-Mora J.L. (2016). Glutamate microbiosensors based on Prussian Blue modified carbon fiber electrodes for neuroscience applications: In-vitro characterization. Sens. Actuators B Chem..

[150] Batra B., Yadav M., Pundir C.S. (2016). l-Glutamate biosensor based on l-glutamate oxidase immobilized onto ZnO nanorods/polypyrrole modified pencil graphite electrode. Biochem. Eng. J..

[151] Cui Y., Barford J.P., Renneberg R. (2007). Development of an interference-free biosensor for l-glutamate using a bienzyme salicylate hydroxylase/l-glutamate dehydrogenase system. Enzyme Microb. Technol..

[152] Cui Y., Barford J.P., Renneberg R. (2007). Development of an l-glutamate biosensor using the coimmobilization of l-glutamate dehydrogenase and p-hydroxybenzoate hydroxylase on a Clark-type electrode. Sens. Actuators B Chem..

[153] Jamal M., Xu J., Razeeb K.M. (2010). Disposable biosensor based on immobilisation of glutamate oxidase on Pt nanoparticles modified Au nanowire array electrode. Biosens. Bioelectron..

[154] Hughes G., Pemberton R.M., Fielden P.R., Hart J.P. (2017). A reagentless, screen-printed amperometric biosensor for the determination of glutamate in food and clinical applications. Methods Mol. Biol..

[155] Batra B., Pundir C.S. (2013). An amperometric glutamate biosensor based on immobilization of glutamate oxidase onto carboxylated multiwalled carbon nanotubes/gold nanoparticles/chitosan composite film modified Au electrode. Biosens. Bioelectron..

[156] Yu H., Ma Z., Wu Z. (2015). Immobilization of Ni-Pd/core-shell nanoparticles through thermal polymerization of acrylamide on glassy carbon electrode for highly stable and sensitive glutamate detection. Anal. Chim. Acta.

[157] Batra B., Kumari S., Pundir C.S. (2014). Construction of glutamate biosensor based on covalent immobilization of glutmate oxidase on polypyrrole nanoparticles/polyaniline modified gold electrode. Enzyme Microb. Technol..

[158] Singh R., Kashyap S., Kumar S., Abraham S., Gupta T.K., Kayastha A.M., Malhotra B.D., Saxena P.S., Srivastava A., Singh R.K. (2017). Excellent storage stability and sensitive detection of neurotoxin quinolinic acid. Biosens. Bioelectron..

[159] Fritea L., Tertiș M., Cosnier S., Cristea C., Săndulescu R. (2015). ELECTROCHEMICAL SCIENCE A Novel Reduced Graphene Oxide/β-Cyclodextrin/Tyrosinase Biosensor for Dopamine Detection. Int. J. Electrochem. Sci..

[160] Taly A., Corringer P.J., Guedin D., Lestage P., Changeux J.P. (2009). Nicotinic receptors: Allosteric transitions and therapeutic targets in the nervous system. Nat. Rev. Drug Discov..

[161] Lozier B.K., Haven T.R., Astill M.E., Hill H.R. (2015). Detection of acetylcholine receptor modulating antibodies by flow cytometry. Am. J. Clin. Pathol..

[162] Luchicchi A., Bloem B., Viaña J.N.M., Mansvelder H.D., Role L.W. (2014). Illuminating the role of cholinergic signaling in circuits of attention and emotionally salient behaviors. Front. Synaptic Neurosci..

[163] Colliver T.L., Ewing A.G. (2006). Neurotransmitters, Electrochemical Detection of. Encyclopedia of Analytical Chemistry.

[164] Picollo F., Battiato A., Bernardi E., Marcantoni A., Pasquarelli A., Carbone E., Olivero P., Carabelli V. (2016). Microelectrode Arrays of Diamond-Insulated Graphitic Channels for Real-Time Detection of Exocytotic Events from Cultured Chromaffin Cells and Slices of Adrenal Glands. Anal. Chem..

[165] Lin Y., Trouillon R., Svensson M.I., Keighron J.D., Cans A.S., Ewing A.G. (2012). Carbon-ring microelectrode arrays for electrochemical imaging of single cell exocytosis: Fabrication and characterization. Anal. Chem..

[166] Yakushenko A., Schnitker J., Wolfrum B. (2012). Printed carbon microelectrodes for electrochemical detection of single vesicle release from PC12 cells. Anal. Chem..

[167] Liu X., Tong Y., Fang P.P. (2019). Recent development in amperometric measurements of vesicular exocytosis. TrAC Trends Anal. Chem..

[168] Sangubotla R., Kim J. (2018). Recent trends in analytical approaches for detecting neurotransmitters in Alzheimer’s disease. TrAC Trends Anal. Chem..

[169] Emran M.Y.Y., Mekawy M., Akhtar N., Shenashen M.A.A., EL-Sewify I.M.M., Faheem A., El-Safty S.A.A. (2018). Broccoli-shaped biosensor hierarchy for electrochemical screening of noradrenaline in living cells. Biosens. Bioelectron..

[170] Byrnes K.R., Wilson C.M., Brabazon F., Von Leden R., Jurgens J.S., Oakes T.R., Selwyn R.G. (2014). FDG-PET imaging in mild traumatic brain injury: A critical review. Front. Neuroenergetics.

[171] Taylor I.M., Robbins E.M., Catt K.A., Cody P.A., Happe C.L., Cui X.T. (2017). Enhanced dopamine detection sensitivity by PEDOT/graphene oxide coating on in vivo carbon fiber electrodes. Biosens. Bioelectron..

[172] Yang C., Trikantzopoulos E., Nguyen M.D., Jacobs C.B., Wang Y., Mahjouri-Samani M., Ivanov I.N., Venton B.J. (2016). Laser Treated Carbon Nanotube Yarn Microelectrodes for Rapid and Sensitive Detection of Dopamine in Vivo. ACS Sens..

[173] Park J.W., Bhimani R.V., Park J. (2017). Noradrenergic Modulation of Dopamine Transmission Evoked by Electrical Stimulation of the Locus Coeruleus in the Rat Brain. ACS Chem. Neurosci..

[174] Van Schoors J., Viaene J., Van Wanseele Y., Smolders I., Dejaegher B., Vander Heyden Y., Van Eeckhaut A. (2016). An improved microbore UHPLC method with electrochemical detection for the simultaneous determination of low monoamine levels in in vivo brain microdialysis samples. J. Pharm. Biomed. Anal..

[175] Nguyen T.N.H., Nolan J.K., Park H., Lam S., Fattah M., Page J.C., Joe H.E., Jun M.B.G., Lee H., Kim S.J. (2019). Facile fabrication of flexible glutamate biosensor using direct writing of platinum nanoparticle-based nanocomposite ink. Biosens. Bioelectron..

[176] Xiao G., Xu S., Song Y., Zhang Y., Li Z., Gao F., Xie J., Sha L., Xu Q., Shen Y. (2019). In situ detection of neurotransmitters and epileptiform electrophysiology activity in awake mice brains using a nanocomposites modified microelectrode array. Sens. Actuators B Chem..

[177] Ferreira N.R., Ledo A., Laranjinha J., Gerhardt G.A., Barbosa R.M. (2018). Simultaneous measurements of ascorbate and glutamate in vivo in the rat brain using carbon fiber nanocomposite sensors and microbiosensor arrays. Bioelectrochemistry.

[178] Oh Y., Heien M.L., Park C., Kang Y.M., Kim J., Boschen S.L., Shin H., Cho H.U., Blaha C.D., Bennet K.E. (2018). Tracking tonic dopamine levels in vivo using multiple cyclic square wave voltammetry. Biosens. Bioelectron..

[179] Baker K.L., Bolger F.B., Lowry J.P. (2017). Development of a microelectrochemical biosensor for the real-time detection of choline. Sens. Actuators B Chem..

[180] Baker K.L., Bolger F.B., Lowry J.P. (2015). A microelectrochemical biosensor for real-time in vivo monitoring of brain extracellular choline. Analyst.

[181] Gu H., Liu Y., Ren T., Xia W., Guo Y., Shi G. (2019). An electrochemical biosensor based on double molecular recognition for selective monitoring of cerebral dopamine dynamics at 4 min interval. Sens. Actuators B Chem..

[182] Maduraiveeran G., Sasidharan M., Ganesan V. (2018). Electrochemical sensor and biosensor platforms based on advanced nanomaterials for biological and biomedical applications. Biosens. Bioelectron..

[183] Borisova T., Kucherenko D., Soldatkin O., Kucherenko I., Pastukhov A., Nazarova A., Galkin M., Borysov A., Krisanova N., Soldatkin A. (2018). An amperometric glutamate biosensor for monitoring glutamate release from brain nerve terminals and in blood plasma. Anal. Chim. Acta.

[184] Ganesana M., Trikantzopoulos E., Maniar Y., Lee S.T., Venton B.J. (2019). Development of a novel micro biosensor for in vivo monitoring of glutamate release in the brain. Biosens. Bioelectron..

[185] Zestos A.G., Jacobs C.B., Trikantzopoulos E., Ross A.E., Venton B.J. (2014). Polyethylenimine carbon nanotube fiber electrodes for enhanced detection of neurotransmitters. Anal. Chem..

[186] Wang L., Song Y., Zhang Y., Xu S., Xu H., Wang M., Wang Y., Cai X. (2017). A microelectrode array electrodeposited with reduced graphene oxide and Pt nanoparticles for norepinephrine and electrophysiological recordings. J. Micromech. Microeng..

